# A systematic review and meta-analysis on glymphatic flow dysfunction in Parkinson’s disease and Parkinsonism spectrum

**DOI:** 10.1038/s41531-025-01151-4

**Published:** 2025-10-23

**Authors:** Sadegh Ghaderi, Sana Mohammadi, Ali Fathi Jouzdani, Amir Mahmoud Ahmadzadeh, Farzad Fatehi

**Affiliations:** 1 Neuromuscular Research Center, Department of Neurology, Shariati Hospital, Tehran University of Medical Sciences, Tehran, Iran; 2 School of Cognitive Sciences, Institute for Research in Fundamental Sciences (IPM), Tehran, Iran; 3 Neuroscience & Neoplasia Artificial Intelligence Research Group (NAIRG), Department of Neuroscience, School of Science and Advanced Technologies in Medicine, Hamadan University of Medical Sciences, Hamadan, Iran; 4 Department of Radiology, School of Medicine, Mashhad University of Medical Sciences, Mashhad, Iran

**Keywords:** Parkinson's disease, Parkinson's disease, Neurodegeneration, Diagnostic markers, Neuro-vascular interactions, Transporters in the nervous system, Ion channels in the nervous system, Neurodegeneration, Parkinson's disease

## Abstract

This systematic review and meta-analysis synthesized evidence of glymphatic dysfunction in Parkinson’s disease (PD) and related Parkinsonian syndromes using the diffusion tensor imaging along the perivascular space (DTI-ALPS) index. Following the PRISMA 2020 guidelines, 28 studies published up to May 12, 2025, were included. The primary meta-analysis of 21 studies (1678 patients with PD and 1088 HCs) demonstrated a significant reduction in the DTI-ALPS index in patients with PD (Cohen’s d = −0.57; 95% CI: −0.67 to −0.47; p < 0.001), indicating moderate glymphatic dysfunction. This impairment was significantly associated with clinical progression, including longer disease duration (β = −0.08, p = 0.005) and higher Hoehn and Yahr (H-Y) stages (β = −0.18, p = 0.057). This association was particularly strong in patients with early-stage PD (H-Y < 2.5). Phenotypic analyses revealed a gradient of dysfunction, with the most severe impairment found in patients with PD with dementia (Glass’s Δ = −1.04) and atypical Parkinsonian syndromes (Δ = −1.01). Technical subgroup analyses showed consistent findings, with region-of-interest size significantly moderating effect size (p = 0.04). The methodological quality of the included studies was high, with no evidence of publication bias. The methodological quality was high, with no evidence of publication bias (p ≥ 0.88). These findings underscore the progressive glymphatic decline with PD severity and duration, independent of age or cognition, and highlight distinct impairment patterns across different clinical phenotypes.

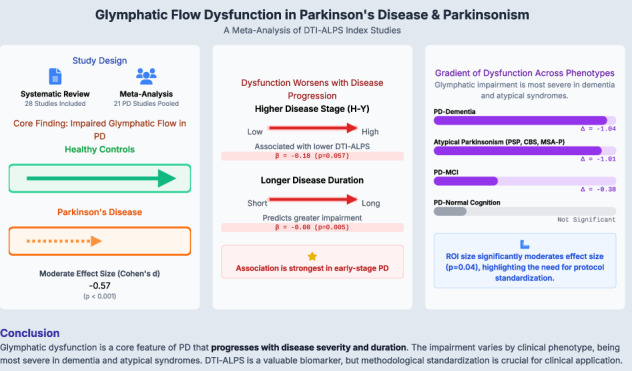

## Introduction

Parkinson’s disease (PD) is a complex neurodegenerative disorder characterized by the progressive degeneration of dopaminergic neurons in the substantia nigra, leading to a decline in dopamine levels, which is essential for motor control^1^. The global prevalence of PD is expected to increase significantly by 2050, primarily because of demographic shifts associated with population aging and extended life expectancy^2^. It is projected to affect approximately 572 per 100,000 individuals over the age of 45 in North America, and it is expected to climb to an estimated 1.2 million in the U.S. alone by 2030^3^. PD involves a continuum of disorders, ranging from idiopathic PD to atypical Parkinsonian syndromes, genetic forms, early onset types, and data- and symptom-based subtypes, each with different clinical characteristics and courses^4^. This heterogeneity is expressed as a progression over a span ranging from a slow decline over two decades or more to an accelerated decline over a few years^5^. Several neuropathological characteristics of PD have been reported, including degeneration of dopaminergic neurons extending over the substantia nigra, formation of Lewy bodies, reduction of dopamine transmitters in the striatum, imbalance between dopamine and acetylcholine transmitters, and iron deposition^6,7^. The cluster motor symptoms of PD are mainly caused by the loss of dopaminergic innervation within the nigrostriatal pathway and involve bradykinesia, rest tremor, rigidity, and postural instability^8^.

The glymphatic pathway, the “Garbage Truck of the Brain”^9^, was discovered through two-photon microscopy examination of the movement of cerebrospinal fluid (CSF) within the live mouse brain in 2012^10,11^. The brain-wide perivascular network that facilitates the exchange of CSF and interstitial fluid (ISF), driven by astrocytic aquaporin-4 (AQP4) channels through perivenous pathways and meningeal lymphatics, plays a pivotal role in maintaining brain health^12–14^. Recent studies, including those in animal models and humans, have indicated that glymphatic pathway dysfunction is related to the pathogenesis of PD^15,16^. For instance, in a transgenic mouse model of PD, blocking meningeal lymphatic drainage aggravates PD-like pathology^17^. Similarly, progressive degeneration of dopaminergic neurons may exacerbate impairment of the glymphatic pathway system^18^. Understanding the glymphatic function offers new insights into the development of neurodegenerative diseases, particularly those characterized by progressive dopaminergic degeneration, such as PD^13^.

**Fig. 1:**
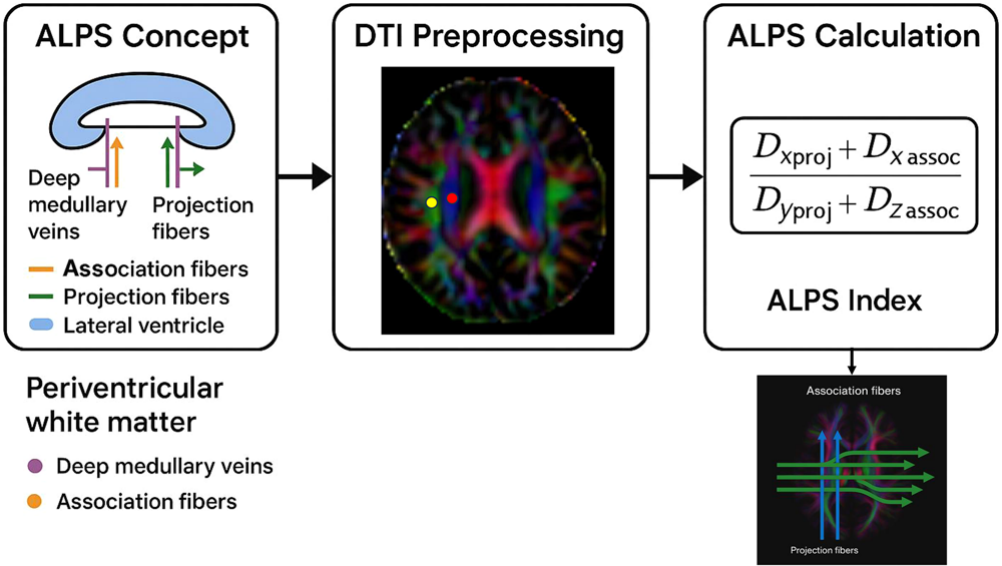
Schematic illustration of the Along the Perivascular Space (ALPS) index methodology. D represents mean diffusivity along specified axes.

The in vivo assessment of the glymphatic system in humans presents significant challenges, prompting the development of various imaging techniques to non-invasively probe its function^19,20^. Although methods such as dynamic contrast-enhanced MRI (DCE-MRI) can track the movement of intrathecally administered tracers, providing direct evidence of fluid flow, their invasive nature limits their widespread clinical application^21^. Consequently, several non-invasive MRI techniques have been adapted to serve as proxies of glymphatic activity. These include phase-contrast MRI to measure CSF pulsatility, intravoxel incoherent motion (IVIM) to quantify microvascular and interstitial fluid movement, and advanced diffusion models to disentangle free-water contributions^19,20^. Among these, the diffusion tensor imaging along the perivascular space (DTI-ALPS) index has recently emerged as a practical and widely adopted method^22^. This technique specifically quantifies the diffusivity of water along the orientation of deep medullary veins, providing an indirect in vivo surrogate measure of perivascular clearance activity and overall glymphatic system function in the brain (Fig. 1).

The DTI-ALPS index, which quantifies diffusivity along the deep medullary vein at the level of the lateral ventricle body, enables in vivo assessment of perivascular clearance activity and glymphatic system function in the brain^22^. Previous research has established that the DTI-ALPS index mediates the interaction between the tTau/β-amyloid1-42 ratio^23^ and changes in cognition^24^, and that it is closely related to the severity of PD^25,26^ and motor symptoms^27^. Furthermore, lower diffusivity within perivascular spaces, represented by a decreased ALPS index, reflects glymphatic dysfunction within PD, and its relationship with increased plasma nuclear DNA levels suggests an increase in oxidative stress accompanying these microstructural changes^28^. Specifically, sleep disturbances, most notably insomnia, obstructive sleep apnea, and rapid eye movement (REM) sleep behavior disorder, correlate with decreased glymphatic function in patients with PD, as indicated by diminished DTI-ALPS values^29–31^. Furthermore, disrupted glymphatic clearance is responsible for non-motor symptoms such as mental decline, depression, and anxiety^32^. In addition, neuroimaging helps differentiate the glymphatic system of PD from essential tremors, further solidifying its status as an α-synucleinopathy^33,34^. Comparable glymphatic deficits have been observed in associated diseases, such as multiple system atrophy, Parkinsonian type (MSA-P), and corticobasal degeneration (CBD), highlighting the wider application of ALPS as a cross-condition imaging agent^35^.

Thus, our systematic review and meta-analysis aimed to (1) synthesize evidence on glymphatic dysfunction in patients with PD and related Parkinsonism syndromes compared to healthy controls (HCs), (2) evaluate the magnitude of glymphatic impairment across distinct clinical phenotypes, including PD-dementia (PD-D), PD-mild cognitive impairment (PD-MCI), PD-normal cognition (PD-NC), and other Parkinsonism syndromes, and (3) investigate the moderating roles of demographic, clinical, and technical factors (e.g., disease stage and imaging protocols) in the observed heterogeneity. By establishing glymphatic dysfunction as a key process in PD, these findings could pave the way for using DTI-ALPS as a biomarker to monitor novel, non-dopaminergic therapies aimed at enhancing glymphatic clearance, such as sleep optimization or pharmacological modulation of aquaporin-4 channels, thereby opening new avenues for disease-modifying strategies in PD.

## Results

### Overview of results

**Fig. 2:**
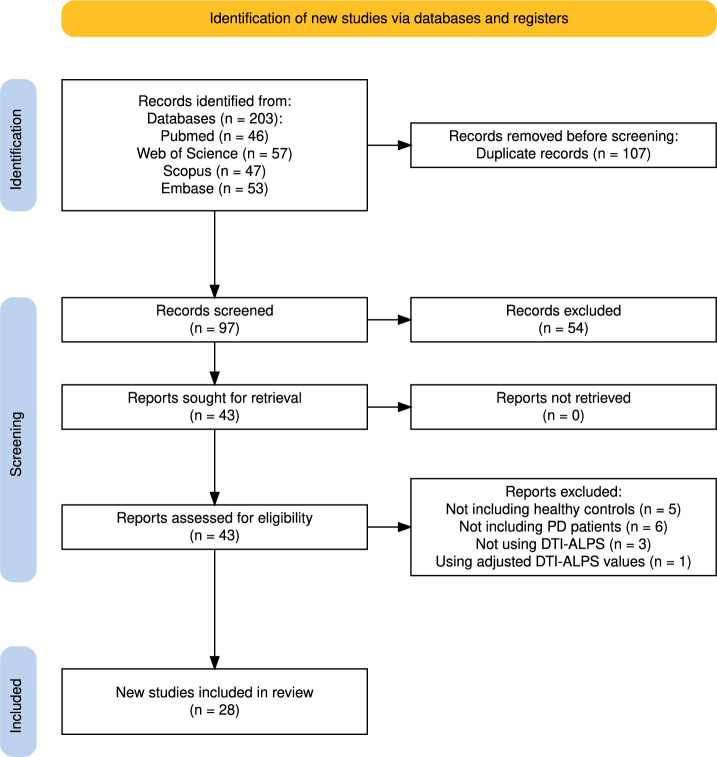
PRISMA flow diagram illustrating study identification, screening, and inclusion (PD, Parkinson’s Disease; DTI-ALPS, Diffusion Tensor Imaging Along the Perivascular Space).

A systematic search of major databases up to May 12, 2025, yielded 203 records (Supplementary Table 1). After removing 107 duplicates, 97 studies were screened, of which 54 were excluded. Of the 43 full-text articles assessed, 13 were excluded due to the absence of HCs (n = 5), lack of PD cohorts (n = 6), non-use of DTI-ALPS (n = 3), and adjusted DTI-ALPS metrics (n = 1). Ultimately, 28 studies were included^23,25,27–30,32,33,36–55^ (Fig. 2). Demographic, clinical, cognitive, neuropsychological, and imaging data were extracted. Relevant data were extracted from eligible studies on PD and other Parkinsonian forms (Supplementary Tables 2 and 3).

The main meta-analysis pooled data from 21 studies^23,25,27,29,30,32,33,36,38–44,48–50,53–55^ (n = 1678 PD patients, n = 1088 HCs) to compare glymphatic function (DTI-ALPS index) between patients with PD and HCs. Subgroup analyses were conducted for age, coil configuration, diffusion direction, and sex distribution (Table 1). Meta-regression analyses were performed for demographic, clinical, and neuropsychological predictors (Table 2). Beyond the main pooled PD vs. HC comparison, several other pooled meta-analyses were conducted, including (1) PD-D, (2) PD-MCI, (3) PD-NC, and (4) other Parkinsonism syndromes (progressive supranuclear palsy (PSP), CBS, and MSA-P) (Table 3).

**Table 1:** Summary of subgroup analysis results: Parkinson’s Disease (PD) vs. Healthy Controls (HC)

Variables	k	Cohen’s d (95% CI)	Heterogeneity χ2	p-value	I² (%)	Group Differences p-value
Age						
Age ≤ 64	13	−0.61 (−0.75, −0.47)	24.25	0.02	51.25	0.30
Age > 64	8	−0.50 (−0.64, 0.36)	3.24	0.86	0	
Sex Distribution						
Male % > 50	15	−0.57 (−0.70, −0.44)	26	0.01	47.05	0.94
Male % ≤ 50	6	−0.58 (−0.73, −0.43)	2.89	0.72	0.00	
MRI Coil Configuration						
Coil < 32	8	−0.52 (−0.66, −0.38)	9.19	0.24	24.84	0.09
Coil ≥ 32	6	−0.73 (−0.93, −0.53)	5.93	0.31	18.73	
Diffusion Directions						
Directions ≤ 32	11	−0.57 (−0.68, −0.45)	9.72	0.47	0.00	0.98
Directions > 32	7	−0.56 (−0.79, −0.34)	15.94	0.03	63.13	
DTI Acquisition Scheme						
Single-shell	15	−0.61 (−0.73, −0.49)	22.29	0.07	37.66	0.19
Multi-shell	6	−0.48 (−0.63, −0.32)	4.63	0.46	0.00	
Center of Data Acquisition						
Single-center	17	−0.58 (−0.68, −0.49)	19.07	0.26	6.68	0.72
Multi-center	4	−0.53 (−0.81, −0.25)	9.49	0.02	67.90	
ROI Placement Method						
Manual	18	−0.60 (−0.71, −0.49)	25.76	0.08	33.80	0.1
Atlas-based	3	−0.40 (−0.61, −0.18)	0.19	0.91	0.00	
ROI Size						
ROI = 5 mm	11	−0.59 (−0.72, −0.47)	13.19	0.21	28.99	0.04
ROI < 5 mm	5	−0.37 (−0.54, −0.20)	1.23	0.87	0.00	
ROI Shape						
Spheres/Circular	17	−0.57 (−0.67, −0.47)	19.16	0.26	21.37	0.77
Cube/Rectangular	2	−0.48 (−1.10, 0.14)	3.34	0.07	70.1

**Table 2:** Meta-regression results of clinical progression

Model/Predictors	β (SE)	p-value	95% CI	R² (%)	Heterogeneity (I²%)	k
Disease duration, MoCA				100.00	0.00	**7**
Disease duration (year)	−0.08 (0.03)	0.008	[−0.13, −0.02]			
MoCA	−0.05 (0.04)	0.258	[−0.13, 0.04]			
Disease duration, Age, MoCA				100.00	0.00	**7**
Disease duration (year)	−0.08 (0.03)	0.005	[−0.14, −0.02]			
Age (year)	0.02 (0.02)	0.406	[−0.03, 0.06]			
MoCA	−0.04 (0.04)	0.344	[−0.13, 0.04]			
H-Y < 2.5 (early PD)	−0.30 (0.13)	0.022	[−0.55, −0.04]	100.00	0.00	13
H-Y overall	−0.18 (0.09)	0.057	[−0.37, 0.005]	70.07	8.30	16

All models used REML estimation. Residual homogeneity tests were non-significant (Q_res p > 0.30). Beta (β) values represent standardized coefficients (Cohen’s d).

*MoCA* Montreal Cognitive Assessment, *H-Y* Hoehn and Yahr stage.

**Table 3:** Summary of results of other main pooled Parkinsonian subtype meta-analyses

Meta-analysis	k	Cohen’s d (CI 95%)	z	p-value	Heterogeneity χ^2^	I^2^	p-value _heterogeneity_
PD	21	−0.57 (−0.67, −0.47)	−11.45	<0.001	28.90	30.78	0.09
PD-normal cognition	3	−0.20 (−0.67, 0.27)	−0.84	0.40	4.65	56.55%	0.10
PD with mild cognitive impairment	4	−0.38 (−0.63, −0.12)	−2.89	<0.001	1.74	0.00%	0.63
PD-Dementia	3	−1.04 (−1.36, −0.72)	−6.31	<0.001	1.20	0.00%	0.55
Other Parkinsonism disorders	6	−1.01 (−1.69, 0.33)	−2.90	<0.001	33.91	82.95%	<0.001

### Main meta-analysis

#### Main pooled meta-analysis: Parkinson’s disease vs. healthy controls

**Fig. 3:**
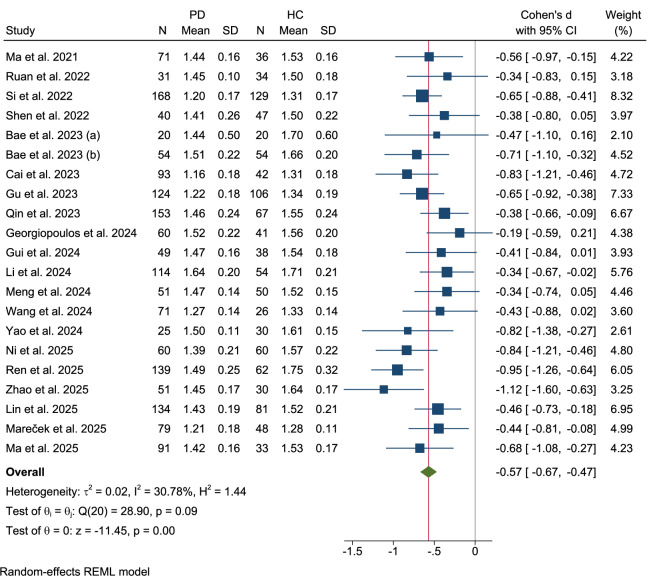
Forest plot of main polled meta-analysis: Parkinson’s disease (PD) vs. healthy controls (HCs).

A random-effects model revealed a statistically significant reduction in the DTI-ALPS index in patients with PD (Cohen’s d = −0.57, 95% CI: −0.67 to −0.47), indicating moderate-to-large standardized mean differences (SMDs) favoring HCs (Fig. 3). Marginally low heterogeneity was observed across the studies (τ²=0.02, I² = 30.78%, H² = 1.44), with a non-significant heterogeneity test (Q(20) = 26.43, p = 0.09). The test for overall effect demonstrated robust significance (z = −11.45, p < 0.001), underscoring consistent glymphatic dysfunction in the PD cohort.

**Fig. 4:**
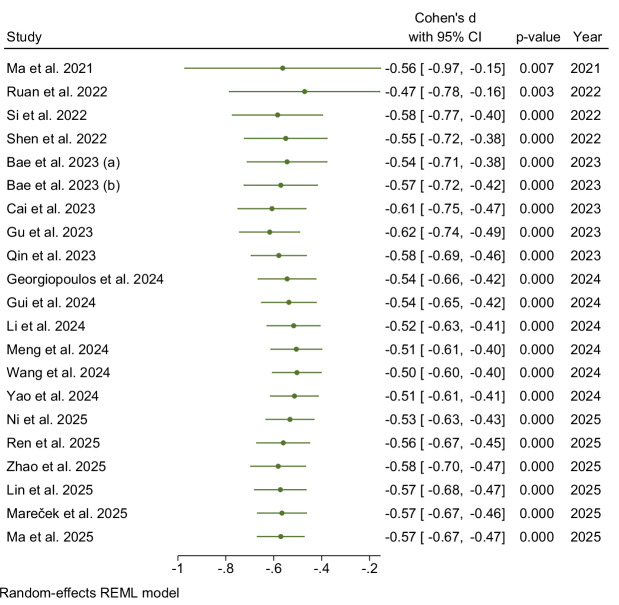
Cumulative forest plot stratified by publication year.

A cumulative meta-analysis ordered by publication year was performed to assess the evolution of the estimated SMD as more data became available (Fig. 4). The analysis began with studies published in 2021, yielding an initial significant Cohen’s d of −0.56 (95% CI: −0.97, −0.15; p = 0.007). As studies from subsequent years were incrementally added, the pooled SMD demonstrated early stabilization of the results. For instance, after the inclusion of studies from 2022, the cumulative Cohen’s d was −0.55 (95% CI: −0.72 to −0.38). Throughout 2023, with the addition of five further studies, the cumulative SMD remained consistently significant and within a narrow range. The incorporation of six studies from 2024 further solidified this trend, with the cumulative Cohen’s d showing minor fluctuations, for example, being −0.51 (95% CI: −0.61, −0.41). The final inclusion of six studies anticipated in 2025 resulted in an overall cumulative Cohen’s d of −0.57 (95% CI: −0.67 to −0.47).

### Age subgroup

**Fig. 5:**
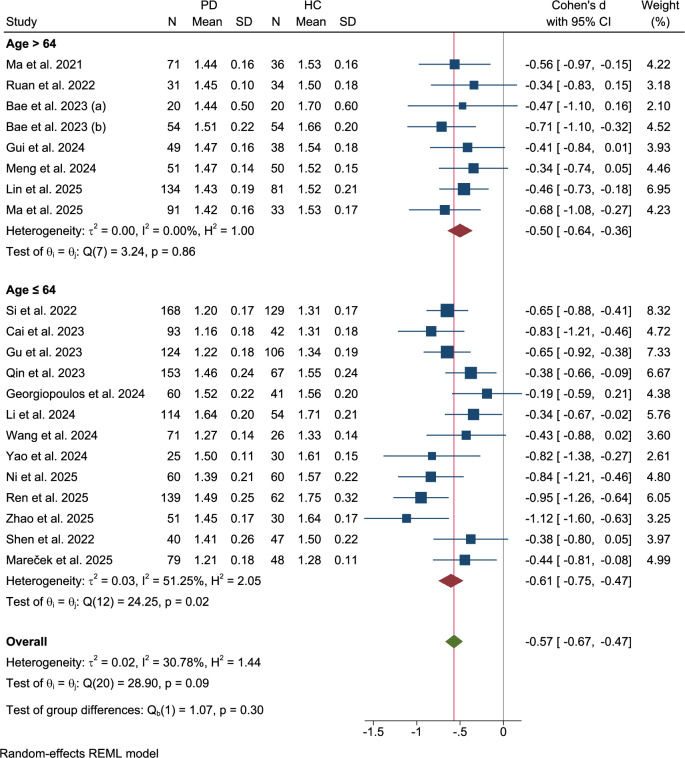
Subgroup analysis: age stratification.

The studies were stratified by the age of patients with PD (cutoff: 64 years) (Fig. 5). For cohorts with a mean age of >64 years (k = 8), glymphatic dysfunction (Cohen’s d = −0.50, 95% CI: −0.64 to −0.36) exhibited homogeneity (τ² = 0.00, I² = 0%, Q(7) = 2.40, p = 0.86). Studies with a mean age ≤64 years (k = 13) showed moderate-to-large SMDs (Cohen’s d = −0.61, 95% CI: −0.75 to −0.47), suggesting consistent further DTI-ALPS reductions in younger PD populations with moderate heterogeneity (τ² = 0.03, I² = 51.25%, Q(12) = 24.25, p = 0.02), despite a similar SMD. No significant differences were observed between the groups (Q = 1.07, p = 0.30).

### Sex subgroup

**Fig. 6:**
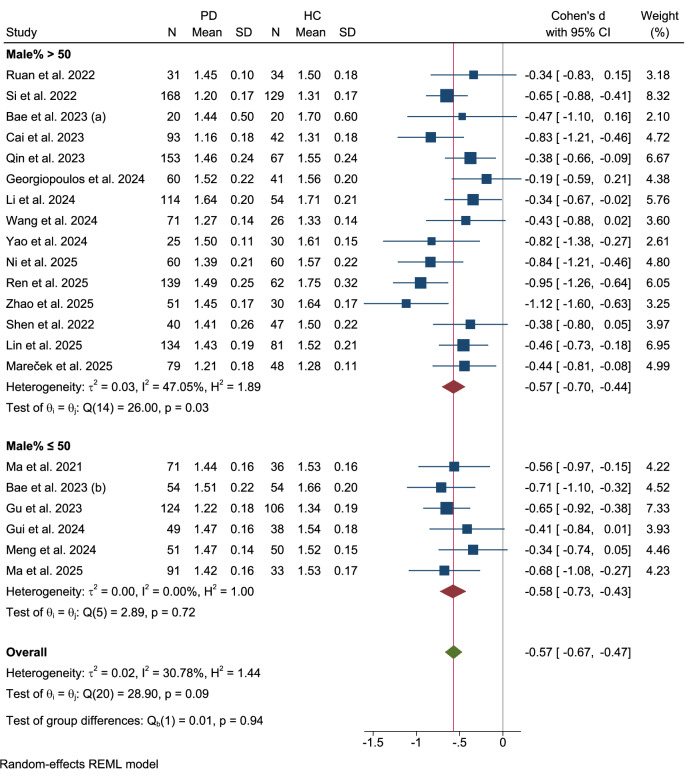
Subgroup analysis: sex distribution.

Stratification by male predominance ( > 50% vs. ≤50% males) (Fig. 6) showed no significant differences between the subgroups (Q = 0.01, p = 0.94). Cohorts with >50% males (k = 15) demonstrated a Cohen’s d of −0.57 (95% CI: −0.70 to -0.44) and moderate heterogeneity (τ²=0.03, I² = 47.05%, Q(14) = 26.00, p = 0.03), while studies with ≤50% males (k = 6) were homogeneous (τ²=0.00, I² = 0.00%) with a nearly similar SMD (Cohen’s d = −0.58, 95% CI: −0.73 to −0.43).

### MRI coil configuration subgroup

**Fig. 7:**
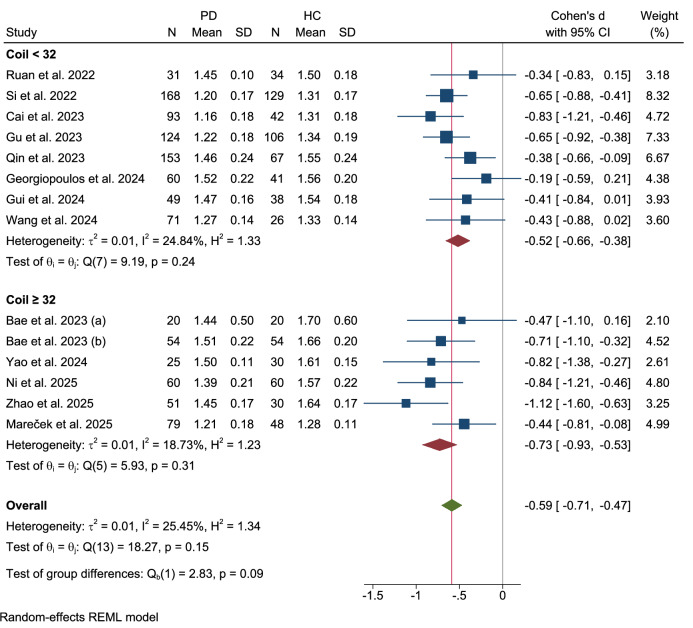
Subgroup analysis: MRI coil configuration.

Stratification by the MRI coil channel number ( < 32 vs. ≥32) (Fig. 7) revealed no significant subgroup differences (Q = 2.83, p = 0.09). Studies using coils with ≥32 channels (k = 6) demonstrated homogeneous effects (τ²=0.01, I² = 18.73%, p = 0.31) with a moderate-to-large mean Cohen’s d of −0.73 (95% CI: −0.93 to -0.53), likely reflecting enhanced imaging precision. Studies with coils with<32 channels (k = 8) showed smaller effects (Cohen’s d = −0.52, 95% CI: −0.66 to −0.38) and low heterogeneity (τ²=0.01, I² = 24.84%, Q(7) = 9.19, p = 0.24).

### DTI diffusion directions and acquisition methods subgroup

**Fig. 8:**
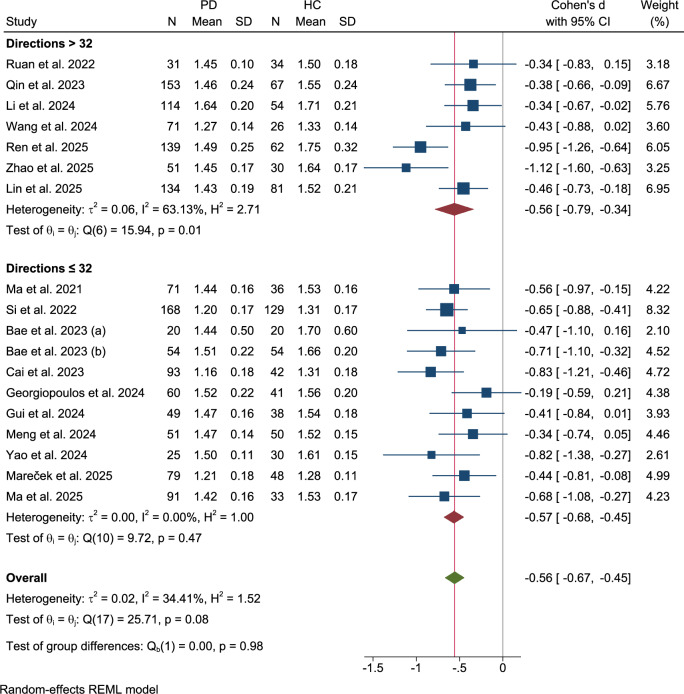
Subgroup analysis: DTI diffusion directions.

Subgrouping by the number of diffusion directions ( > 32 vs. ≤32) (Fig. 8) revealed no significant group differences (Q = 0.00, p = 0.98). Studies employing >32 directions (k = 6) exhibited a Cohen’s d of −0.56 (95% CI: −0.79 to −0.34), with high heterogeneity (τ²=0.0, I² = 63.13%, Q(5) = 15.94, p = 0.01), whereas studies with ≤32 directions (k = 9) showed low homogeneity (τ²=0.00, I² = 00.00%, Q(8) = 9.72, p = 0.47) and a approximately same Cohen’s d of −0.57 (95% CI: −0.68 to −0.45).

**Fig. 9:**
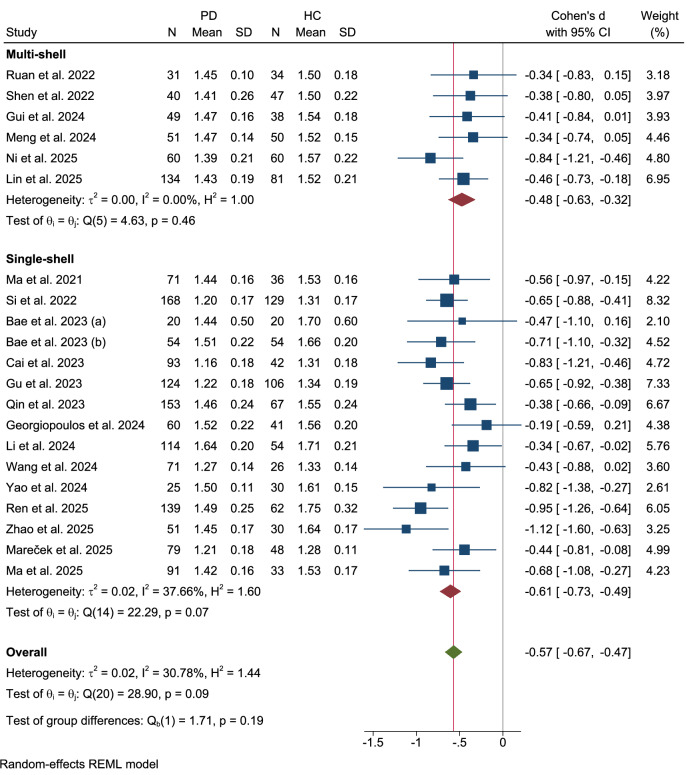
Subgroup analysis by DTI acquisition scheme.

No significant differences were observed between multi-shell and single-shell imaging protocols (Qb(1) = 1.71, p = 0.19) (Fig. 9). The six studies employing a multi-shell technique demonstrated a significant reduction in the DTI-ALPS index in patients with PD (Cohen’s d = −0.48, 95% CI: −0.63 to −0.32), with no evidence of heterogeneity (τ² = 0.00, I² = 0.00%). Similarly, the fifteen studies using a single-shell acquisition showed a comparable and significant effect (Cohen’s d = −0.61, 95% CI: −0.73 to −0.49), with moderate heterogeneity (τ² = 0.02, I² = 37.66%).

### Center of data acquisition

**Fig. 10:**
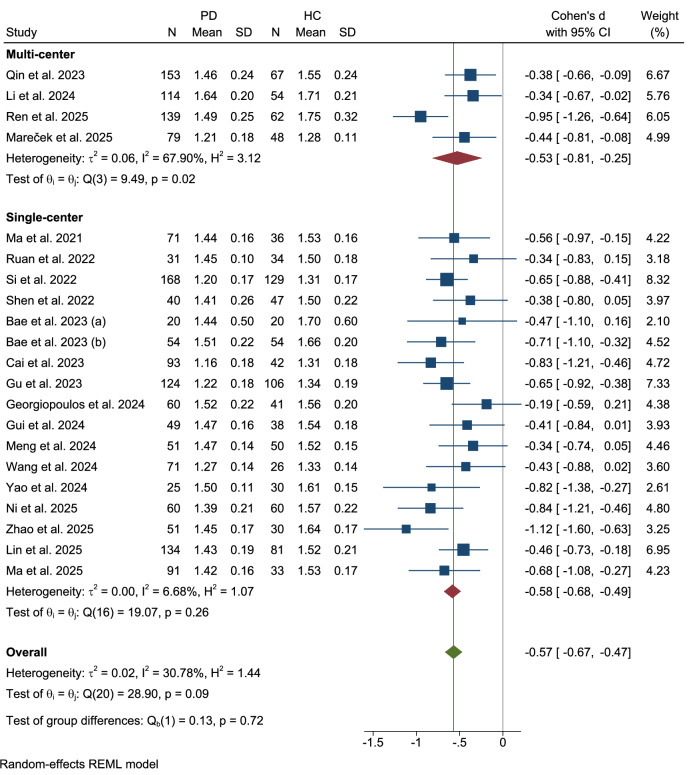
Subgroup analysis by center of data acquisition.

To evaluate the potential impact of inter-site variability, studies were stratified based on whether they were conducted at a single center or multiple centers (Fig. 10). The analysis revealed no significant difference between the two groups (Qb(1) = 0.13, p = 0.72). The four multi-center studies produced a significant pooled effect size (Cohen’s d = −0.53, 95% CI: −0.81 to −0.25), albeit with high heterogeneity (τ² = 0.06, I² = 67.90%). Similarly, the seventeen single-center studies demonstrated a significant effect (Cohen’s d = −0.58, 95% CI: −0.68 to -0.49) with low heterogeneity (τ² = 0.00, I² = 6.68%).

### ROI methodologies subgroup

**Fig. 11:**
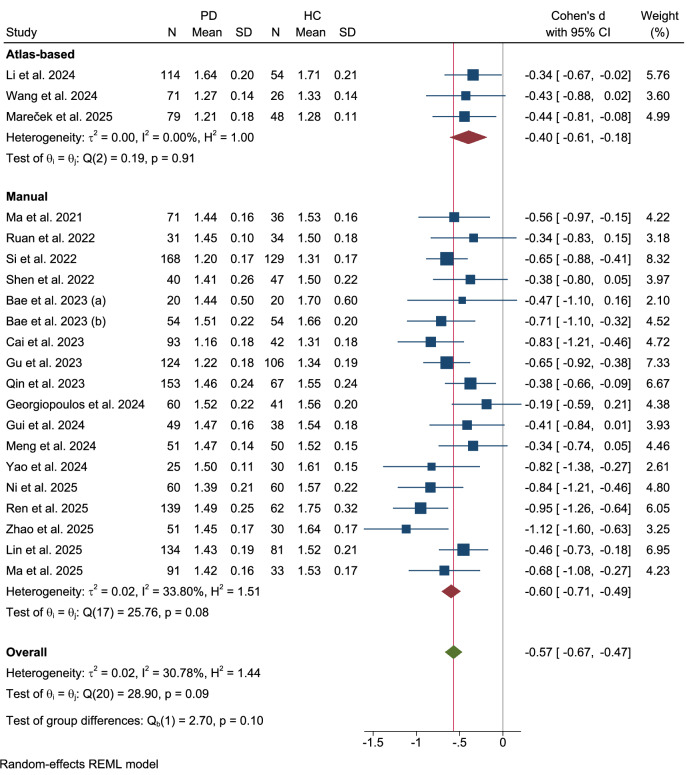
Subgroup analysis by ROI placement method.

**Fig. 12:**
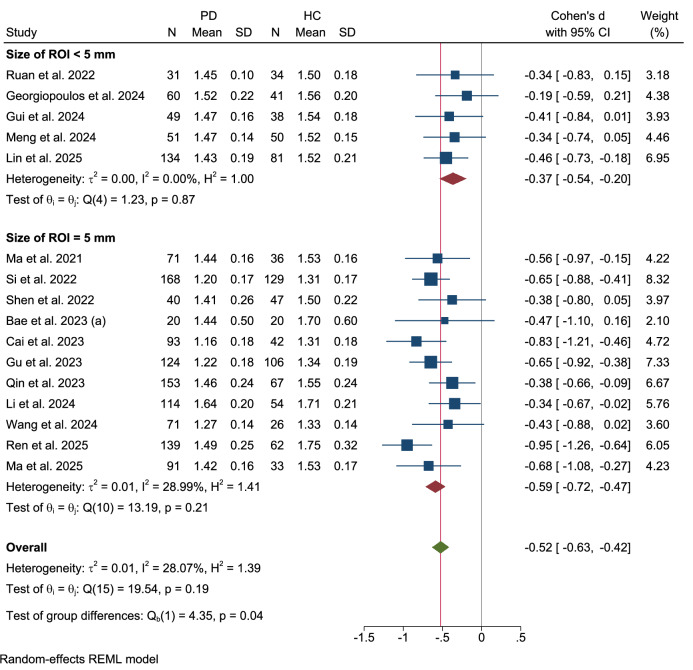
Subgroup analysis by ROI size.

**Fig. 13:**
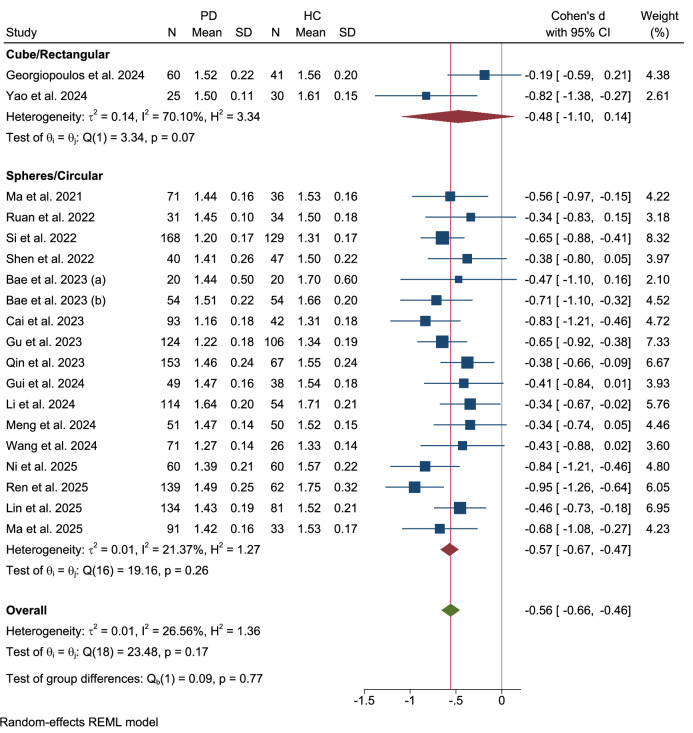
Subgroup analysis by ROI shape.

Further subgroup analyses were conducted to examine the influence of different methodologies for defining regions of interest (ROI) (Figs. 11–13). When stratified by the ROI placement method, no significant difference was observed between studies using an atlas-based approach and those using a manual approach (Qb(1) = 2.70, p = 0.10). The three studies employing an atlas-based method found a significant reduction in the DTI-ALPS index (Cohen’s d = −0.40, 95% CI: −0.61 to −0.18) with no heterogeneity. In contrast, the eighteen studies using manual ROI placement demonstrated a larger effect size (Cohen’s d = −0.60, 95% CI: −0.71 to −0.49) with moderate heterogeneity (τ² = 0.02, I² = 33.80%).

The analysis was also stratified by the size of the ROI used. A significant difference was identified between studies utilizing an ROI size of less than 5 mm versus those employing an ROI of exactly 5 mm (Qb(1) = 4.35, p = 0.04). The five studies with an ROI size smaller than 5 mm showed a smaller but significant effect (Cohen’s d = −0.37, 95% CI: −0.54 to −0.20) with no heterogeneity. Conversely, the eleven studies that utilized a 5 mm ROI reported a larger effect size (Cohen’s d = −0.59, 95% CI: −0.72 to −0.47) with low heterogeneity (τ² = 0.01, I² = 28.99%).

Finally, when comparing ROI shapes, no significant difference was detected between studies that used cube/rectangular ROIs and those that employed spherical/circular ROIs (Qb(1) = 0.09, p = 0.77). The two studies utilizing a cube or rectangular shape showed a significant effect (Cohen’s d = −0.48, 95% CI: −1.10 to 0.14) with high heterogeneity (τ² = 0.14, I² = 70.10%). In contrast, the seventeen studies employing spherical or circular ROIs demonstrated a robust and significant effect (Cohen’s d = −0.57, 95% CI: −0.67 to −0.47) with low heterogeneity (τ² = 0.01, I² = 21.37%).

### Meta-regression of Disease Severity and Duration

**Fig. 14:**
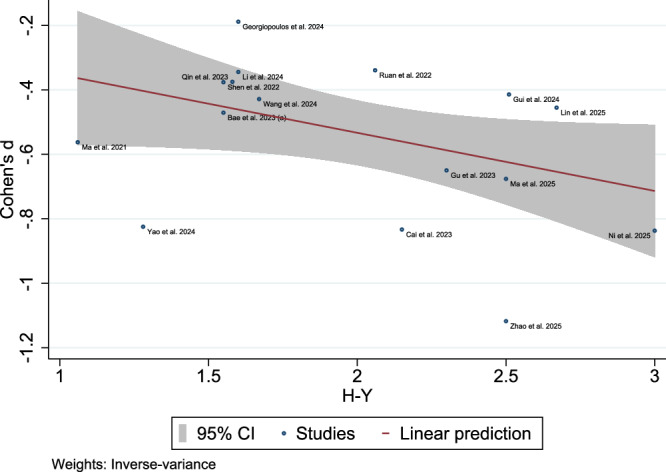
Meta-regression bubble plot results: association with Hoehn and Yahr (H-Y) progression: Association between H-Y stages and glymphatic dysfunction in overall Parkinson’s disease cohorts (k = 16 studies).

**Fig. 15:**
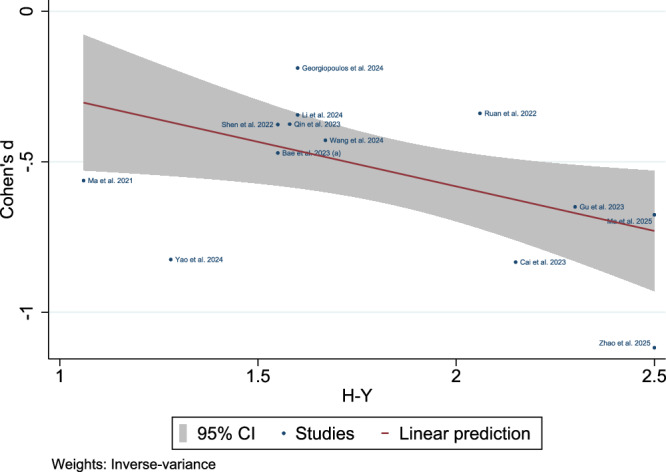
Meta-regression bubble plot results: association with Hoehn and Yahr (H-Y) progression: Significant inverse correlation between H-Y stages and glymphatic dysfunction in early Parkinson’s disease (H-Y < 2.5; k = 13 studies).

**Fig. 16:**
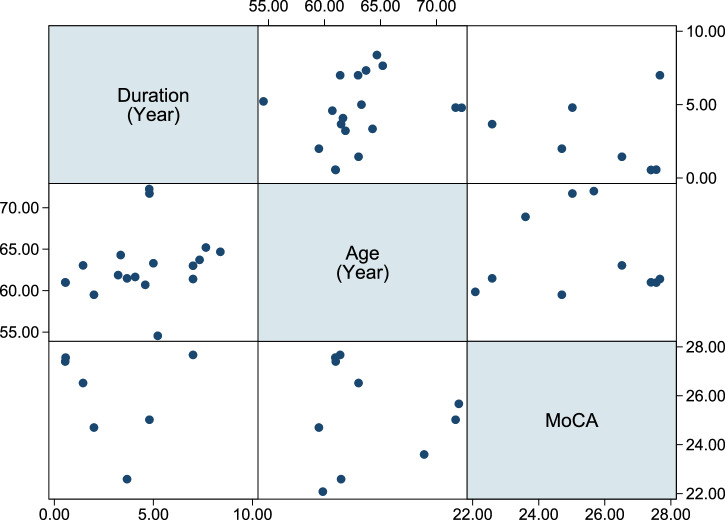
Meta-regression of disease duration, age, and MoCA with glymphatic dysfunction (DTI-ALPS), highlighting a significant negative association with disease duration.

Meta-regression analyses revealed significant associations between glymphatic dysfunction and clinical parameters in patients with PD. For the overall PD cohorts (k = 16 studies that reported Hoehn and Yahr (H-Y) stage), higher H-Y stages exhibited a marginal negative association with SMDs (Cohen’s d: β = −0.18, p = 0.057), explaining 70.07% of the variance (R²) with low residual heterogeneity (I² = 8.30%, tau²=0.0035) (Fig. 14). In early PD (H-Y < 2.5; k = 13), this association was significantly strengthened (β = −0.30, p = 0.022), with a full variance explanation (R² = 100%) and negligible heterogeneity (I² = 0.00%) (Fig. 15). Disease duration independently predicted reduced glymphatic function in models adjusted for age and Montreal Cognitive Assessment (MoCA) (β = −0.08, p = 0.005; k = 7) and in a reduced model excluding age (β = −0.08, p = 0.008; k = 7), although age and MoCA showed no significant effects (p > 0.25) (Fig. 16). All models utilized REML estimation, demonstrated non-significant residual heterogeneity (Q_res p > 0.30), and robust Wald tests (p < 0.05 for H-Y < 2.5 and duration models). These results underscore the progressive glymphatic impairment with advancing PD severity and disease duration, independent of age and cognitive status.

### Pooled meta-analysis: PD with Dementia (PD-Dementia) vs. healthy controls

**Fig. 17:**
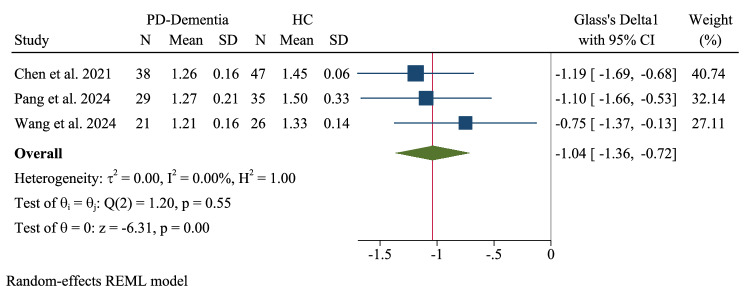
Pooled meta-analysis: Parkinson’s disease (PD)-dementia vs. healthy controls (HCs).

Three PD-D studies demonstrated a significant reduction in glymphatic function compared to HCs (Glass’s Δ1 = −1.04, 95% CI: −1.36 to −0.72) (Fig. 17). Notably, this analysis exhibited homogeneity (τ²=0.00, I² = 0%, H² = 1.00) with no significant heterogeneity (Q(2) = 1.20, p = 0.55) and a highly significant overall effect (z = −6.31, p < 0.001).

### Pooled meta-analysis: PD with mild cognitive impairment (PD-MCI) vs. healthy controls

**Fig. 18:**
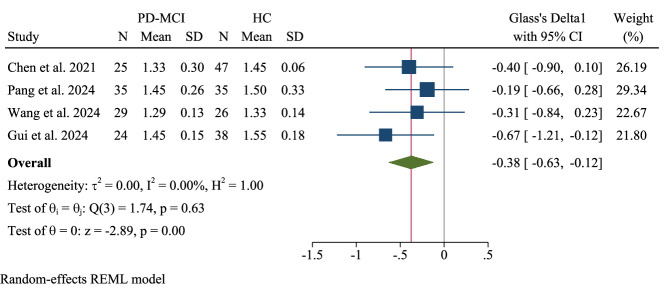
Pooled meta-analysis: Parkinson’s disease (PD) with mild cognitive impairment (PD-MCI) vs. healthy controls (HCs).

Four studies of patients with PD-MCI revealed a smaller yet statistically significant reduction in the DTI-ALPS index (Glass’s Δ1 = −0.38, 95% CI: −0.63 −0.12) (Fig. 18). The analysis showed no heterogeneity (τ²=0.00, I² = 0%, H² = 1.00; Q(3) = 1.74, p = 0.63), with a significant overall effect (z = −2.89, p = 0.004).

### Pooled meta-analysis: PD with normal cognition (PD-NC) vs. healthy controls

**Fig. 19:**
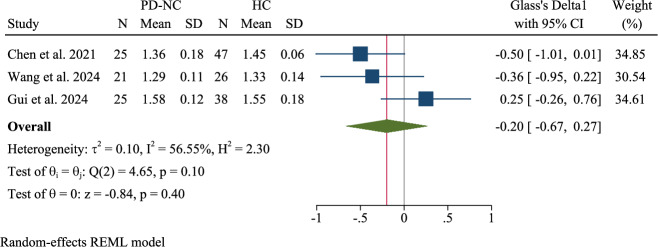
Pooled meta-analysis: Parkinson’s disease (PD)with normal cognition (PD-NC) vs. healthy controls (HCs).

Three studies of patients with PD-NC found no statistically significant difference in the DTI-ALPS index compared to HCs (Glass’s Δ1 = −0.20, 95% CI: -0.67 to 0.27) (Fig. 19). Moderate heterogeneity was observed (τ²=0.10, I² = 56.55%, H² = 2.30; Q(2) = 4.65, p = 0.10), although the overall effect was not significant (z = −0.84, p = 0.40), suggesting that the glymphatic function may remain relatively preserved in patients with PD without cognitive deficits.

### Pooled meta-analysis: other Parkinsonism syndromes vs. healthy controls

**Fig. 20:**
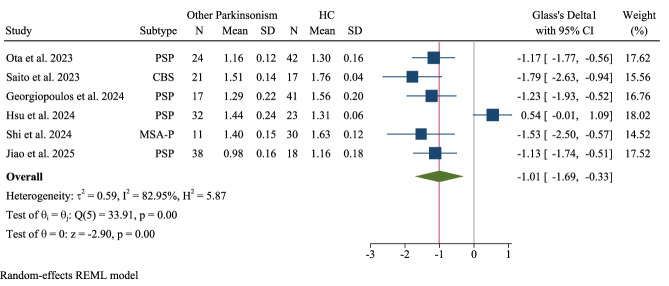
Pooled meta-analysis: other Parkinsonism syndromes (progressive supranuclear palsy (PSP), corticobasal syndrome (CBS), multiple system atrophy (MSA-P) vs. healthy controls (HCs).

Six studies involving patients with PSP, CBS, or MSA-P demonstrated substantial glymphatic dysfunction (Glass’s Δ1 = −1.01, 95% CI: −1.69 to −0.33) (Fig. 20). However, significant heterogeneity was observed (τ²=0.59, I² = 82.95%, H² = 5.87; Q(5) = 33.91, p < 0.001), likely reflecting the clinical and pathological diversity across Parkinsonian subtypes. The overall effect remained robust (z = −2.90, p = 0.004), indicating severe glymphatic impairment in atypical Parkinsonian disorder patients.

### Quality and publication bias assessment

**Fig. 21:**
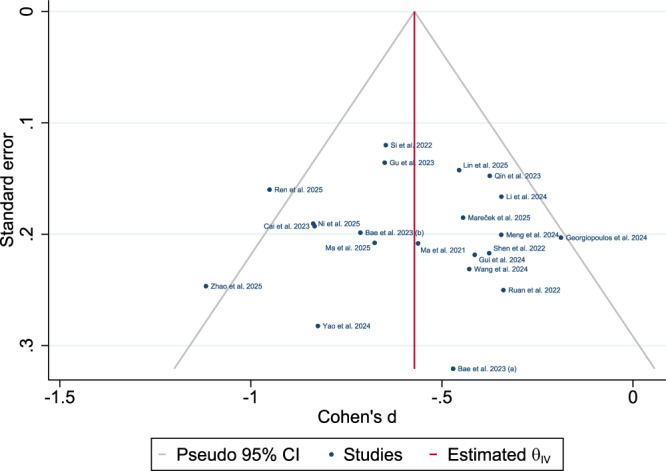
Funnel plot for publication bias assessment.

**Fig. 22:**
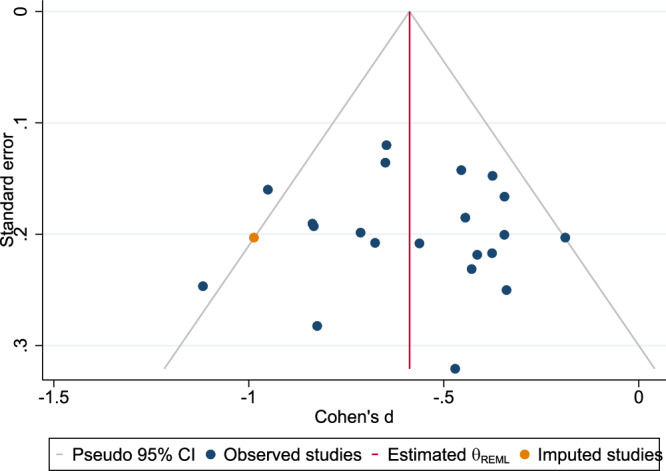
Trim-and-fill adjusted funnel plot (left-side imputation).

The risk of bias assessment (Supplementary Table 4) indicated high methodological quality across the studies. The funnel plot symmetry supported minimal bias, confirming the robustness of the meta-analysis findings (Fig. 21). Publication bias analyses also revealed no evidence of small-study effects using Egger’s (β = 0.12, p = 0.99) or Begg’s (p = 0.88) tests. Trim-and-fill analysis imputed one missing study, yielding a negligible SMD shift (observed: Cohen’s d = −0.57 vs. adjusted: −0.59) (Fig. 22).

### Sensitivity analysis

**Fig. 23:**
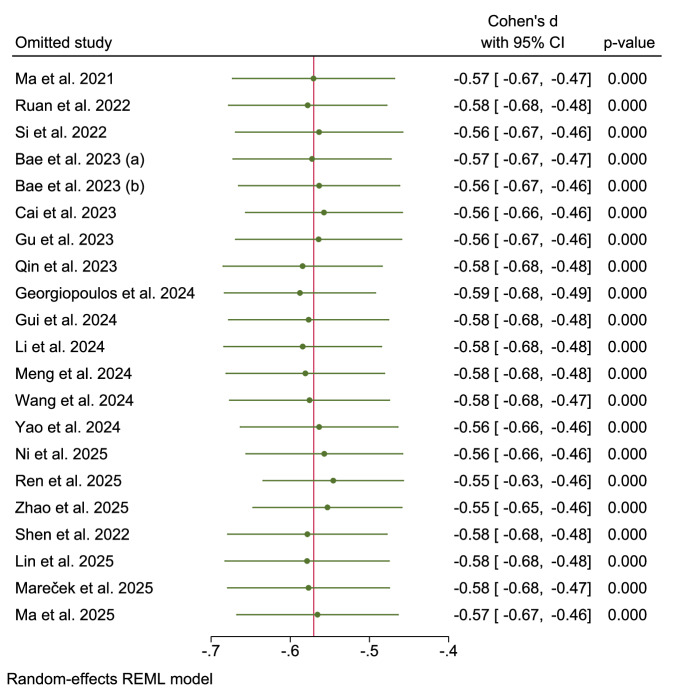
Sensitivity analysis: Leave-One-Out.

Leave-one-out sensitivity analysis demonstrated the robust stability of the pooled SMD (Cohen’s d range: −0.55 to −0.59) when the individual studies were sequentially excluded (Fig. 23). The overall effect of glymphatic dysfunction (DTI-ALPS index reduction in PD vs. HCs) remained statistically significant across all iterations, with minimal variability in magnitude (Δ Cohen’s d = 0.04). The narrow confidence intervals and consistent significance confirmed that no single study disproportionately influenced the meta-analysis results.

## Discussion

Today, several dMRI techniques^13,56–58^, including low b-value diffusion-weighted imaging^59^, optimized b-value sampling for dMRI^60^, IVIM^61,62^, and DCE-MRI for tracking tracer movement^10^, phase-contrast MRI, and ultrafast encephalography measure pulsatile CSF^63,64^ as well as advanced dMRI models, such as diffusion analysis, neurite orientation dispersion and density imaging for disentangling free-water contributions (NODDI)^65,66^, diffusion kurtosis imaging (DKI)^67^, and FW indices^68^, have also been developed. Beyond all of these, recent developments have introduced DTI-ALPS^22^ to measure diffusion anisotropy, while intrathecal MRI demonstrates only minor CSF influx into the deep white matter^69^, opening new windows into CSF fluid dynamics.

Our DTI-ALPS findings align with and extend those of numerous individual studies reporting glymphatic dysfunction in PD and Parkinsonism^24,26,70–73^. Critically, glymphatic dysfunction progresses with clinical severity and disease duration, independent of age or cognitive status. These results underscore the potential role of the glymphatic system in PD pathophysiology and its relevance as a biomarker for staging and subtyping neurodegenerative processes.

The glymphatic system has been conceptualized as a brain-wide perivascular waste clearance pathway, and multiple lines of evidence have implicated its failure in the pathogenesis of proteinopathies. Notably, animal experiments have shown that glymphatic flux contributes to α-synuclein clearance: suppression of perivascular flow (e.g., via AQP4 knockout) retards α-syn removal and accelerates synucleinopathy in PD models^34,74^. Thus, our meta-analytic observation of reduced ALPS in PD is consistent with the hypothesis that impaired ISF clearance promotes pathological α-synuclein accumulation^11,17,75,76^.

While a prior meta-analysis^73^ established initial evidence for glymphatic impairment in Parkinson’s disease by pooling 11 studies, the present work offers a more comprehensive and updated synthesis. By including 28 studies, our analysis provides enhanced statistical power and, more importantly, delves deeper by (1) evaluating glymphatic function across distinct clinical and cognitive phenotypes (e.g., PD with dementia, mild cognitive impairment, and normal cognition), (2) assessing atypical Parkinsonian syndromes, and (3) performing detailed meta-regressions to investigate the association between glymphatic impairment and moderators, such as disease severity and duration. These expanded analyses are crucial for elucidating the role of the glymphatic system in the pathophysiology and progression of Parkinson’s disease.

The observed glymphatic impairment may reflect multiple interacting mechanisms^13,77,78^, including PVS enlargement due to impaired ISF drainage, astrocytic dysfunction disrupting AQP4 polarization, and neurovascular decoupling reducing pulsatile arterial drives for glymphatic flow. Furthermore, the noradrenergic system, which is known to be compromised in PD, plays a critical role in modulating glymphatic clearance through its control of neurovascular coupling and arousal states, adding another layer to the potential pathophysiology^35^.

The glymphatic pathway normally supports the exchange of CSF-ISF along the PVSs, helping to wash out soluble metabolites (including amyloid-β, tau, and α-synuclein)^13,79–81^. When this exchange diminishes, neurotoxic proteins may accumulate^16^. In PD, a failure to clear extracellular α-synuclein could exacerbate Lewy-body formation and dopaminergic cell loss^6,36,82^. Indeed, glymphatic blockade has been reported to exacerbate motor and memory deficits in PD models^83^. Conversely, protein aggregates may obstruct perivascular channels^84,85^. This bidirectional link suggests that PD may represent a form of “interstitial fluidopathy”^86^, wherein abnormal solute dynamics contribute to disease progression. Our finding that glymphatic dysfunction was most pronounced in cognitively impaired PD patients also contradicts the notion that clearance deficits may underlie cortical neurodegeneration^74,87^, in line with evidence that sleep disturbances impair glymphatic flow and promoting dementia^48^. This parallels Alzheimer’s disease, in which glymphatic transport is thought to influence amyloid-β and tau burdens^88^. The gradient of effect sizes (PD-D > PD-MCI > PD-NC) suggests that glymphatic impairment may worsen with cognitive decline, possibly reflecting spreading pathology or advancing AQP4 mislocalization^38,46,89^. Early detection of glymphatic impairment may inform therapeutic interventions aimed at enhancing clearance, such as sleep optimization or targeted CSF circulation therapies^90^. Additionally, the absence of significant impairment in the PD-NC group indicates a potential window for intervention before the manifestation of cognitive deficits. Although causality cannot be inferred here, these results raise the possibility that reduced interstitial flow contributes to the risk of developing dementia in patients with PD.

Patients with PSP, CBS, and MSA-P exhibit profound dysfunction of the glymphatic system. However, significant heterogeneity across these studies likely reflects the diverse neuropathologies underlying atypical Parkinsonism tauopathy in PSP/CBS versus α-synucleinopathy in MSA-P and their distinct effects on perivascular clearance pathways^91,92^. These results underscore the need for subtype-specific analyses in future studies, as glymphatic impairment may arise through different mechanisms in various Parkinsonian disorders.

Meta-regression analyses identified the H-Y stage and disease duration as independent predictors of glymphatic impairment, with a higher H-Y stage and longer disease duration correlating with reduced DTI-ALPS indices. In early stage PD (H-Y < 2.5), the association between the H-Y stage and glymphatic dysfunction was particularly pronounced, suggesting that glymphatic compromise accelerates during the initial disease phases^35,71^. These results point towards a progressive decline in glymphatic clearance capacity as PD advances^72^ and align with neuropathological models positing that α-synuclein aggregation and neuroinflammation processes^27^, potentially modulated by glymphatic clearance, are already active in early PD^35,93^. Glymphatic impairment may exacerbate α-synuclein aggregation owing to reduced ISF clearance, creating a vicious cycle of neurodegeneration^94^. Therapeutic strategies that enhance glymphatic function, such as sleep optimization or AQP4 modulation, could mitigate this cascade, as suggested by preclinical models^12^. The persistence of this association after adjusting for age and cognitive status (MoCA scores) further implicates glymphatic dysfunction as a core component of PD progression rather than a secondary epiphenomenon.

We tested whether demographic or technical factors moderated the glymphatic deficits. The PD versus HC effect remained significant across the subgroups without any significant group differences. Stratification by age, MRI coil configuration, diffusion direction, and sex distribution revealed no statistically significant subgroup differences, indicating that glymphatic dysfunction in PD is a generalized phenomenon that transcends these variables. However, nuanced trends have emerged: studies utilizing high-channel MRI coils ( ≥ 32 channels) reported larger SMDs than those with lower-resolution systems. This pattern suggests that advanced imaging protocols may enhance the detection sensitivity for glymphatic alterations, which is a critical consideration in future biomarker studies. Similarly, while age stratification showed comparable SMDs between younger ( ≤ 64 years) and older ( > 64 years) PD cohorts, the former exhibited greater heterogeneity, potentially reflecting earlier and more variable glymphatic decline in patients with younger-onset PD.

To further explore the robustness of our findings, we conducted several subgroup analyses based on other key technical and methodological variables. These analyses revealed that the observed glymphatic dysfunction in Parkinson’s disease was largely consistent across different DTI acquisition schemes (multi-shell vs. single-shell), study settings (multi-center vs. single-center), and ROI definition strategies (atlas-based vs. manual; cube/rectangular vs. spherical/circular), as no significant group differences were found in these comparisons. This consistency strengthens the conclusion that the DTI-ALPS index is a reliable marker of glymphatic impairment in PD, independent of these specific methodological choices. However, we did identify a significant moderating effect for ROI size, with studies utilizing a 5 mm ROI reporting a larger effect size than those using smaller ROIs. This suggests that ROI parameterization can influence the magnitude of the detected effect, highlighting the critical need for methodological standardization, particularly regarding ROI dimensions, to improve cross-study comparability and the clinical translation of the DTI-ALPS index.

This study had several limitations that warrant caution. Most studies have been conducted in East Asian populations; therefore, the generalizability of these findings to other ethnicities or milder (earlier) stages of PD remains to be tested. Finally, although publication bias appeared to be minimal, unpublished negative findings may still exist.

The DTI-ALPS index is an indirect surrogate marker of glymphatic flow^95,96^. This technique assumes that the diffusivity along the medullary veins reflects CSF–ISF exchange; however, this assumption has been questioned. For instance, contrast-enhanced MRI studies suggest that parenchymal CSF penetration into the deep white matter is limited, implying that ALPS may not directly measure true glymphatic flux. Thus, our outcome measure should be viewed as a marker of perivascular microstructure or clearance potential, rather than as definitive proof of altered bulk flow.

The minimal impact of MRI parameters (coil configuration and diffusion direction) on the pooled SMDs supports the reliability of DTI-ALPS as a glymphatic biomarker across imaging protocols. However, the trend toward larger SMDs with high-channel coils suggests that technological advancements could enhance the detection of subtle glymphatic changes, particularly in the pre-symptomatic or prodromal PD stages. Standardization of DTI-ALPS acquisition protocols^97,98^-including diffusion direction number, echo time, b-values, and automatic region-of-interest placement remains crucial for cross-study comparability and clinical translation^56,58^. Furthermore, our analysis was constrained by the level of technical detail reported in the primary studies. Factors such as the DTI acquisition scheme (e.g., single-shell vs. multi-shell) and the specific methodology for region-of-interest (ROI) placement (e.g., manual vs. automated, choice of software pipeline) can influence the DTI-ALPS index but were not consistently documented across all included articles. This reporting gap prevented a dedicated subgroup analysis. Future studies should adhere to standardized reporting guidelines to facilitate more precise meta-analyses and clarify the impact of these technical variables on glymphatic assessment.

Future longitudinal and multiparametric studies of DTI-ALPS (or other glymphatic metrics) changes over time in PD and whether baseline ALPS predicts subsequent motor or cognitive decline are needed. Studies on prodromal conditions (e.g., REM sleep behavior disorder) could clarify whether glymphatic impairment precedes clinical PD. Combining DTI-ALPS with other imaging markers, such as the quantitative PVS burden, AQP4 positron emission tomography, or intrathecal contrast MRI, would help validate its meaning^20,99–101^. Interventional studies are also of interest; for example, improving sleep quality or targeting AQP4 polarization may enhance glymphatic clearance and slow the progression of the disease. Moreover, future research should aim to integrate DTI-ALPS with fluid biomarkers. An important next step would be to investigate the relationship between the DTI-ALPS index and CSF concentrations of pathological proteins, such as α-synuclein. Such studies would help validate whether a lower ALPS index is functionally linked to impaired clearance of neurotoxic solutes from the brain interstitium.

Finally, another significant limitation is the potential for unmeasured confounding factors. Glymphatic function is influenced by a wide range of variables, including vascular risk factors (e.g., lipid profile, glucose intolerance), cerebral small vessel disease, sleep quality, and common PD co-morbidities such as depression, anxiety, and autonomic dysfunction^102,103^. However, the majority of the studies included in our meta-analysis did not systematically report or control for these potential confounders, primarily adjusting only for age and sex. This reporting gap prevented us from performing a comprehensive meta-regression to evaluate the impact of these variables on the DTI-ALPS index. Therefore, while our findings are robust, the potential influence of these unaddressed factors cannot be dismissed and highlights the need for future studies with more rigorous control of such variables.

To conclude, this meta-analysis provides robust evidence that glymphatic dysfunction is a significant and consistent feature of Parkinsonian disorders. Our findings strongly support the role of glymphatic impairment in PD pathophysiology, progressing alongside clinical severity (H-Y stage) and disease duration, with an accelerated decline observed in early-stage PD. The analysis highlights distinct patterns across phenotypes, with severe dysfunction in PD-Dementia and atypical Parkinsonian syndromes, in contrast to the relatively preserved glymphatic activity in cognitively intact patients. Furthermore, these findings remained robust across most demographic, technical, and methodological subgroups, emphasizing the core role of clinical progression. However, the significant moderating effect of ROI size highlights the need for standardized imaging protocols to enhance cross-study comparability. While acknowledging the inherent limitations of the DTI-ALPS index as an indirect measure, these findings underscore its potential as a valuable biomarker for disease staging and phenotypic differentiation, offering important insights for future therapeutic strategies targeting glymphatic pathways in PD.

## Methods

### Search strategy and selection process

This review and meta-analysis were conducted in strict accordance with the PRISMA 2020 guidelines^104^. Until May 12, 2025, we performed comprehensive searches of PubMed, Web of Science, Scopus, and Embase databases to identify studies assessing glymphatic dysfunction in PD and related Parkinsonian disorders using the DTI-ALPS index. Our search strategy was framed by the PECO model (Population: PD and Parkinsonism patients; Exposure: DTI-ALPS; Comparator: HCs; Outcome: ALPS index), combined MeSH terms and keywords for glymphatic function, neuroimaging techniques, and disease processes using Boolean operators to optimize sensitivity and specificity (Supplementary Table 1). We tailored each database’s syntax to ensure maximal retrieval of relevant studies. We included original peer-reviewed articles that quantitatively compared ALPS indices between affected patients and HCs, excluding non-human research, abstracts, reviews, editorials, preprints, and any reports lacking numerical ALPS data. The retrieved records were imported into the Rayyan platform (https://www.rayyan.ai/) and EndNote software (v.21) for deduplication and management. Two independent reviewers (A.A. and A.F.) screened the titles and abstracts, followed by a full-text evaluation. Disagreements were resolved by consensus with senior investigators (S.G. and S.M.) and chief professor (F.F.) to mitigate selection bias.

### Data extraction

Data were extracted in a structured manner by four reviewers (A.A., A.F., S.G., and S.M.) employing a uniform data collection template to record the following variables: author and year of publication; DTI acquisition details (including field strength, echo time (TE), number of diffusion-encoding directions, and b-values), participant demographics (age, sex), clinical and cognitive measures (e.g., MoCA, Mini-Mental State Examination (MMSE), Unified Parkinson’s Disease Rating Scale (UPDRS), H-Y stage, and disease duration), and reported ALPS index values. In instances where ALPS indices were not directly provided in the text, values were obtained from graphical representations using the GetData Graph Digitizer (https://getdata-graph-digitizer.software.informer.com/) and PlotDigitizer (https://plotdigitizer.com/app).

### Quality and publication bias assessment

The methodological quality assessment of included studies was evaluated using a modified version of the Newcastle–Ottawa Scale (NOS) adapted for cross-sectional research^105,106^. This instrument appraises bias across three principal domains: (1) selection, addressing representativeness of the sample and adequacy of its size; (2) comparability, examining whether analyses appropriately controlled for key confounders, such as age and sex; and (3) outcome assessment, focusing on the validity of the ALPS index measurement and the clarity of statistical reporting. Two reviewers (A.A. and A.F.) independently assigned NOS scores from 0 (lowest quality) to 9 (highest quality), with discrepancies resolved by consensus, and final ratings categorized as “very good” (9), “good” (7–8), “satisfactory” (5–6), or “unsatisfactory” (0–4)^107^. The senior investigators re-evaluated all scores to ensure consistency. To assess publication bias, funnel plots were generated to detect asymmetry suggestive of selective reporting, and formal tests—including Egger’s regression and Begg’s rank correlation—were conducted^108,109^. The trim-and-fill procedure was further applied to estimate and correct for potential bias, with a significance threshold set at p < 0.05.

### Statistical analysis

Statistical analyses were performed using a random-effects model to accommodate anticipated differences in SMDs across studies. SMDs were interpreted according to Cohen’s benchmarks^110^: 0.2 for small, 0.5 for medium, and 0.8 for large effects^111^ for the main pooled meta-analysis and subgroup analyses, as well as Glass’s delta (Δ) 1 for other pooled analyses^112,113^. Between-study heterogeneity was quantified with the I² statistic and classified as low (0–25%), moderate (25–50%), high (50–75%), or very high (75–100%)^114,115^. We conducted overall and subgroup meta-analyses to examine potential sources of ALPS index variability, and assessed the stability of pooled estimates via leave-one-out sensitivity analyses. We also conducted a meta-regression to explore the impact of demographic, clinical, and neuropsychological predictors on the effect sizes. When necessary, data were transformed to reduce methodological discrepancies using the Meta-Converter platform (https://meta-converter.com/) in line with Cochrane Handbook recommendations^116^. All statistical procedures were executed in STATA version 17.0 (StataCorp, College Station, TX, USA), with two-tailed p values below 0.05 considered statistically significant.

## Supplementary information

Supplementary information

## Data Availability

This article contains all the data produced or analyzed during this investigation. Further inquiries should be forwarded to the corresponding author.
