# Supplementary material for: A systematic review and meta-analysis on glymphatic flow dysfunction in Parkinson’s disease and Parkinsonism spectrum
Source: NPJ Parkinsons Dis. 2025 Oct 23;11:306. doi: 10.1038/s41531-025-01151-4 (PMC12550064; doi:10.1038/s41531-025-01151-4)
Supplement: Supplementary file 1 — Supplementary information [file 41531_2025_1151_MOESM1_ESM.docx]

| **Supplementary Table 1.** The search strategies used for database searches (05/12/2025). This table presents the full electronic search strategies applied to PubMed, Scopus, Web of Science, and Embase for the systematic review. Search terms included combinations of keywords related to glymphatic function, Parkinson’s disease, parkinsonism spectrum disorders, and diffusion tensor imaging along the perivascular space (DTI-ALPS). The column “N” indicates the number of records retrieved from each database prior to deduplication. | | |
| --- | --- | --- |
| **Database** | **Terms** | **N** |
| PubMed | ("glymphatic*"[Title/Abstract]) AND ("parkinson*"[Title/Abstract] OR "progressive supranuclear palsy"[Title/Abstract] OR "multiple system atrophy"[Title/Abstract] OR "corticobasal degeneration"[Title/Abstract]) AND ("DTI-ALPS" [Title/Abstract] OR "ALPS-index" [Title/Abstract] OR "ALPS index" [Title/Abstract] OR "'diffusion tensor image analysis along the perivascular space*" [Title/Abstract] OR "'diffusion tensor imaging analysis along the perivascular space*" [Title/Abstract] OR "diffusion*"[Title/Abstract]) | 46 |
| Scopus | TITLE-ABS ( "glymphatic*" ) AND TITLE-ABS ( ( "parkinson*" OR "progressive supranuclear palsy" OR "multiple system atrophy" OR "corticobasal degeneration" ) AND ( "DTI-ALPS" OR "ALPS-index" OR "ALPS index" OR "diffusion tensor image analysis along the perivascular space*" OR "diffusion tensor imaging analysis along the perivascular space*" OR "diffusion*" ) ) | 47 |
| Web of Science (WOS) | TS=("Glymphatic*") AND TS=("parkinson*" OR "progressive supranuclear palsy" OR "multiple system atrophy" OR "corticobasal degeneration") AND TS=("DTI-ALPS" OR "ALPS-index" OR "ALPS index" OR "diffusion tensor image analysis along the perivascular space*" OR "diffusion tensor imaging analysis along the perivascular space*" OR "Diffusion*") | 57 |
| Embase | ('glymphatic*':ab,ti) AND ('parkinson*':ab,ti OR 'progressive supranuclear palsy':ab,ti OR 'multiple system atrophy':ab,ti OR 'corticobasal degeneration':ab,ti) AND ('DTI-ALPS':ab,ti OR 'ALPS-index':ab,ti OR 'ALPS index':ab,ti OR 'diffusion tensor image analysis along the perivascular space*':ab,ti OR 'diffusion tensor imaging analysis along the perivascular space*':ab,ti OR 'Diffusion*':ab,ti) | 53 |

| **Supplementary Table 2.** Summary of included studies assessing glymphatic flow dysfunction in Parkinson’s disease and parkinsonism spectrum using diffusion tensor imaging along the perivascular space. This table summarizes the main characteristics of the included studies, including MRI field strength, coil type, b-values, number of diffusion directions, participant demographics, clinical subtypes, disease severity measures (e.g., UPDRS, H–Y stage, MoCA, MMSE), disease duration, and DTI-ALPS index values for patients and healthy controls. Abbreviations: Abbreviations: DTI = Diffusion Tensor Imaging; ALPS = Analysis along the Perivascular Space; dTV II.13 = diffusion Tensor Visualizer, version II.13; MRtrix/MRtrix3 = Magnetic Resonance Tractography (version 3); SPM/SPM12 = Statistical Parametric Mapping (version 12); FSL = FMRIB Software Library; DTI Studio = Diffusion Tensor Imaging Studio (Johns Hopkins University); Eddymotion = Eddy-current and motion correction (FSL tool); MATLAB = Matrix Laboratory (MathWorks); ExploreDTI = Explore Diffusion Tensor Imaging; DSI Studio = Diffusion Spectrum Imaging Studio; DTI-TK = Diffusion Tensor Imaging ToolKit; QSM = Quantitative Susceptibility Mapping; Snakemake = Workflow management system for reproducible pipelines; ICBM = International Consortium for Brain Mapping; JHU-ICBM Atlas = Johns Hopkins University – International Consortium for Brain Mapping Atlas; JHU atlas = Johns Hopkins University White Matter Tract Atlas; ROI = Region of Interest; SCR = Superior Corona Radiata; SLF = Superior Longitudinal Fasciculus; SUF = Superior Uncinate Fasciculus; CST = Corticospinal Tract; ICC = Intraclass Correlation Coefficient; 3D Slicer = open-source medical image analysis software; PD = Parkinson’s disease; PSP = Progressive Supranuclear Palsy; CBS = Corticobasal Syndrome; MSA-P = Multiple System Atrophy – Parkinsonian type; PD-MCI = Parkinson’s Disease with Mild Cognitive Impairment; PD-NC = Parkinson’s Disease with Normal Cognition; PD-FOG = Parkinson’s Disease with Freezing of Gait; NR = Not Reported; NA = Not Applicable. | | | | | | | | | | | | | | | | | | | | | | | | | | | | |
| --- | --- | --- | --- | --- | --- | --- | --- | --- | --- | --- | --- | --- | --- | --- | --- | --- | --- | --- | --- | --- | --- | --- | --- | --- | --- | --- | --- | --- |
| **Author/year** | **Field Strength (Tesla)** | **Coil** | **Scanner / DTI Acquisition Parameters** | **b-Value (s/mm²)** | **Directions** | **ROI Method** | **Source of Patient Data** | **Healthy Controls** | | **Patients** | | | | | | | | | | | | | **Patients** | | | **HCs** | | |
|  |  |  |  |  |  |  |  | **Subjects (male)** | **Age (SD)** | **Subtype** | **Patients (male)** | **Age (SD)** | **UPDRS I** | **UPDRS II** | **UPDRS III** | **UPDRS IV** | **UPDRS V** | **UPRDS Total** | **H-Y** | **MOCA** | **MMSE** | **Duration (Year)** | **DTI-ALPS (SD)** | **Alps L (SD)** | **Alps R (SD)** | **DTI-ALPS (SD)** | **HCs Alps L (SD)** | **HCs Alps R (SD)** |
| Bae et al. 2023 (a) | 3 | 32 | Single-shell | 1000 | 32 | Semi-automated preprocessing (DTI-ALPS analyzer) + manual ROI marking (5 mm spheres, bilateral, projection & association fibers) | Single-center (Seoul National University Bundang Hospital, South Korea) | 20 (12) | 73 (19.26) | PD | 20 (12) | 72 (23.70) | NR | NR | 16 (9.57) | NR | NR | NR | 1.55 (0.60) | 25.67 (3.19) | 26.00 (4.79) | NR | 1.44 (0.50) | NR | NR | 1.70 (0.6) | NR | NR |
| Chen et al. 2021 | 3 | NR | Single-shell | 1000 | 13 | Semi-automated preprocessing (dTV II.13) + manual ROI marking (5 mm spheres, left hemisphere, projection & association fibers + subcortical) | Single-center (Kaohsiung Chang Gung Memorial Hospital, Taiwan) | 47 (15) | 61.53 (4.75) | PD-NC | 25 (15) | 60.08 (10.07) | 3.16 (2.29) | 8.40 (6.31) | 19.56 (13.14) | NA | NA | 31.12 (20.26) | 1.86 (1.25) | NR | 27.92 (1.78) | 2.49 (1.91) | 1.36 (0.18) | NR | NR | 1.45 (0.06) | NR | NR |
| Chen et al. 2021 | 3 | NR | Single-shell | 1000 | 13 | Semi-automated preprocessing (dTV II.13) + manual ROI marking (5 mm spheres, left hemisphere, projection & association fibers + subcortical) | Single-center (Kaohsiung Chang Gung Memorial Hospital, Taiwan) | 47 (15) | 61.53 (4.75) | PD-MCI | 25 (8) | 63.80 (8.70) | 3.76 (3.00) | 9.48 (7.50) | 24.76 (15.41) | NA | NA | 38.00 (24.25) | 2.16 (1.26) | NR | 27.08 (1.32) | 3.07 (2.67) | 1.33 (0.30) | NR | NR | 1.45 (0.06) | NR | NR |
| Chen et al. 2021 | 3 | NR | Single-shell | 1000 | 13 | Semi-automated preprocessing (dTV II.13) + manual ROI marking (5 mm spheres, left hemisphere, projection & association fibers + subcortical) | Single-center (Kaohsiung Chang Gung Memorial Hospital, Taiwan) | 47 (15) | 61.53 (4.75) | PD-Dementia | 38 (11) | 65.76 (8.00) | 3.76 (3.07) | 11.55 (8.17) | 27.47 (14.47) | NA | NA | 42.79 (23.98) | 1.88 (0.84) | NR | 20.00 (4.53) | 3.64 (4.28) | 1.26 (0.16) | NR | NR | 1.45 (0.06) | NR | NR |
| Ota et al. 2023 | 3 | NR | Single-shell | 1000 | 15 | Semi-automated preprocessing (Mango software + MRtrix/SPM pipeline) + manual ROI marking (5 mm spheres, bilateral, projection & association fibers) | Single-center (University of Tsukuba, Japan) | 42 (24) | 72.3 (6.1) | PSP | 24 (15) | 72.2 (6.7) | NR | NR | NR | NR | NR | NR | NR | NR | NR | 7.0 (4.0) | 1.16 (0.12) | NR | NR | 1.30 (0.16) | NR | NR |
| Meng et al. 2024 | 3 | NR | Multi-shell | 1000, 2000 | 30 | Semi-automated preprocessing (FSL) + manual ROI marking (4 mm spheres, bilateral, projection & association fibers) | Single-center (Suzhou Hospital affiliated to Nanjing Medical University, China) | 50 (24) | 70.88 (8.00) | PD | 51 (24) | 72.25 (7.82) | 3.67 (2.41) | 14.00 (6.58) | 25.94 (10.02) | NA | NA | 43.61(12.23) | NR | NR | NR | 4.79 (3.58) | 1.47 (0.14) | 1.46 (0.15) | 1.47 (0.13) | 1.52 (0.15) | 1.52 (0.14) | 1.52 (0.16) |
| Meng et al. 2024 | 3 | NR | Multi-shell | 1000, 2000 | 30 | Semi-automated preprocessing (FSL) + manual ROI marking (4 mm spheres, bilateral, projection & association fibers) | Single-center (Suzhou Hospital affiliated to Nanjing Medical University, China) | 50 (24) | 70.88 (8.00) | PD-Early | 29 (15) | 69.34 (7.11) | 2.17 (1.36) | 9.10 (2.94) | 18.45 (4.86) | NA | NA | 29.72 (5.84) | H-Y ≤ 2.5 | NR | NR | 2.93 (1.90) | 1.49 (0.11) | 1.48 (0.15) | 1.49 (0.06) | 1.59 (0.15) | 1.52 (0.14) | 1.66 (0.16) |
| Meng et al. 2024 | 3 | NR | Multi-shell | 1000, 2000 | 30 | Semi-automated preprocessing (FSL) + manual ROI marking (4 mm spheres, bilateral, projection & association fibers) | Single-center (Suzhou Hospital affiliated to Nanjing Medical University, China) | 50 (24) | 70.88 (8.00) | PD-Late | 22 (9) | 76.09 (7.15) | 5.64 (2.04) | 20.45 (3.86) | 35.82 (5.27) | NA | NA | 61.91 (6.85) | H-Y > 2.5 | NR | NR | 7.25 (3.80) | 1.43 (0.15) | 1.42 (0.15) | 1.44 (0.14) | 1.59 (0.15) | 1.52 (0.14) | 1.66 (0.16) |
| Cai et al. 2023 | 3 | 8 | Single-shell | 1000 | 25 | Semi-automated preprocessing (DTI Studio) + manual ROI marking (5 mm spheres, bilateral, projection & association fibers) | Single-center (Guangdong Provincial People’s Hospital, China) | 42 (19) | 61.52 (7.54) | PD | 93 (49) | 61.87 (8.52) | NR | NR | 35.04 (12.52) | NA | NA | NR | 2.15 (0.37) | NR | 27.33 (3.01) | 3.22 (2.91) | 1.16 (0.18) | NR | NR | 1.31 (0.18) | NR | NR |
| Cai et al. 2023 | 3 | 8 | Single-shell | 1000 | 25 | Semi-automated preprocessing (DTI Studio) + manual ROI marking (5 mm spheres, bilateral, projection & association fibers) | Single-center (Guangdong Provincial People’s Hospital, China) | 42 (19) | 61.52 (7.54) | PD-Young | 53 (26) | 55.94 (5.74) | NR | NR | 35.23 (12.81) | NA | NA | NR | 2.15 (0.37) | NR | 27.00 (3.81) | 3.62 (3.31) | 1.22  (0.16) | NR | NR | 1.31 (0.18) | NR | NR |
| Cai et al. 2023 | 3 | 8 | Single-shell | 1000 | 25 | Semi-automated preprocessing (DTI Studio) + manual ROI marking (5 mm spheres, bilateral, projection & association fibers) | Single-center (Guangdong Provincial People’s Hospital, China) | 42 (19) | 61.52 (7.54) | PD-Old | 40 (22) | 69.73 (4.02) | NR | NR | 34.80 (12.30) | NA | NA | NR | 2.15 (0.37) | NR | 27.33 (3.08) | 2.69 (2.20) | 1.09  (0.18) | NR | NR | 1.31 (0.18) | NR | NR |
| Ni et al. 2025 | 3 | 64 | Multi-shell | 250-1500 | Overall=46 | Semi-automated preprocessing (FSL/SPM + Eddymotion) + manual ROI marking (bilateral, projection & association fibers, circular ROIs) | Single-center (Fujian Medical University Union Hospital, China) | 60 (27) | 62.0 (6.70) | PD | 60 (34) | 63.0 (7.0) | NR | NR | 46.0 (15.70) | NA | NA | NR | 3.0 (0.37) | NR | NR | 7.0 (3.91) | 1.39 (0.21) | 1.40 (0.20) | 1.37 (0.3) | 1.57 (0.22) | 1.55 (0.29) | 1.55 (0.21) |
| Ma et al. 2021 | 3 | NR | Single-shell | 1000 | 31 | Semi-automated preprocessing (DTI Studio) + manual ROI marking (5 mm spheres, projection & association fibers, left hemisphere) | Single-center (Chinese Academy of Medical Sciences, China) | 36 (18) | 62.00 (6.24) | PD | 71 (31) | 64.68 (8.12) | 3.04 (1.93) | 12.79 (4.93) | 30.92 (11.58) | 2.68 (2.46) | 1.06 (0.95) | 50.48 (17.25) | 1.06 (0.95) | NR | 28.03 (2.15) | 8.38 (4.29) | 1.44 (0.16) | NR | NR | 1.53 (0.16) | NR | NR |
| Ma et al. 2021 | 3 | NR | Single-shell | 1000 | 31 | Semi-automated preprocessing (DTI Studio) + manual ROI marking (5 mm spheres, projection & association fibers, left hemisphere) | Single-center (Chinese Academy of Medical Sciences, China) | 36 (18) | 62.00 (6.24) | PD-Early | 35 (17) | 63.57 (8.93) | 2.80 (2.13) | 10.40 (4.31) | 26.31 (9.80) | 2.14 (2.35) | 0.97 (1.04) | 42.63 (14.76) | 0.97 (1.04) | NR | 28.09 (2.47) | 6.86 (4.02) | 1.46 (0.15) | NR | NR | 1.53 (0.16) | NR | NR |
| Ma et al. 2021 | 3 | NR | Single-shell | 1000 | 31 | Semi-automated preprocessing (DTI Studio) + manual ROI marking (5 mm spheres, projection & association fibers, left hemisphere) | Single-center (Chinese Academy of Medical Sciences, China) | 36 (18) | 62.00 (6.24) | PD-Late | 36 (14) | 65.75 (7.23) | 3.28 (1.72) | 15.11 (4.4) | 35.39 (11.54) | 3.19 (2.49) | 1.14 (0.87) | 58.11 (16.19) | 1.14 (0.87) | NR | 27.97 (1.83) | 9.86 (4.06) | 1.42 (0.18) | NR | NR | 1.53 (0.16) | NR | NR |
| Georgiopoulos et al. 2024 | 3 | 20 | Single-shell | 1000 | 30 | Semi-automated preprocessing (MATLAB pipeline) + manual ROI marking (2×2 voxel ROIs, bilateral, projection & association fibers, with and without crossing-fiber adjustment) | Single-center (Skåne University Hospital, Sweden), nested in BioFINDER-1 | 41 (15) | 64.5 (8.4) | PD | 60 (33) | 63.3 (1.5) | NR | NR | 10.7 (8.3)​ | NR | NR | NR | 1.6 (0.7) | NR | NR | 5 (3.8) | 1.52 (0.22) | 1.54 (0.20) | 1.51 (0.23) | 1.56 (0.20) | 1.57 (0.18) | 1.55 (0.21) |
| Georgiopoulos et al. 2024 | 3 | 20 | Single-shell | 1000 | 30 | Semi-automated preprocessing (MATLAB pipeline) + manual ROI marking (2×2 voxel ROIs, bilateral, projection & association fibers, with and without crossing-fiber adjustment) | Single-center (Skåne University Hospital, Sweden), nested in BioFINDER-1 | 41 (15) | 64.5 (8.4) | PSP | 17 (9) | 70.9 (1.5) | NR | NR | 37.8 (16.3) | NR | NR | NR | 3.6 (1.3) | NR | NR | 4.1 (2) | 1.29 (0.22) | 1.27 (0.23) | 1.31 (0.20) | 1.56 (0.20) | 1.57 (0.18) | 1.55 (0.21) |
| Ruan et al. 2022 | 3 | 16 | Multi-shell | 0-2000 | 99 | Semi-automated preprocessing (DTI Studio) + manual ROI marking (4 mm circular ROIs, bilateral, projection & association fibers) | Single-center (Guangzhou First People’s Hospital, China) | 34 (15) | 62.71 (4.12) | PD-FOG | 28 (18) | 66.11 (6.46) | 2.04 (2.03) | 12.01 (6.97) | 31.00 (1.53) | 2.11 (2.67) | NR | NR | 2.43 (0.50) | NR | 25.30 (3.55) | 6.11 (5.23) | 1.47 | NR | NR | 1.54 | NR | NR |
| Ruan et al. 2022 | 3 | 16 | Multi-shell | 2000 | 99 | Semi-automated preprocessing (DTI Studio) + manual ROI marking (4 mm circular ROIs, bilateral, projection & association fibers) | Single-center (Guangzhou First People’s Hospital, China) | 34 (15) | 62.71 (4.12) | PD-nFOG | 31 (17) | 64.29 (8.22) | 0.77 (0.99) | 7.38 (4.32) | 28.4 (1.38) | 0.67 (1.35) | NR | NR | 2.06 (0.51) | NR | 25.42 (4.30) | 3.35 (3.47) | 1.45 | NR | NR | 1.5 | NR | NR |
| Hsu et al. 2024 | NR | NR | Single-shell | 1000 | 64 | Semi-automated preprocessing (ExploreDTI + ICBM Atlas) + atlas-based ROI extraction (projection & association fibers, bilateral, periventricular) | Single-center (Linkou Chang Gung Memorial Hospital, Taiwan) | 23 (9) | 65.6 (7.1) | PSP | 32 (17) | 67.6 (4.91) | NR | NR | 25 (20.18) | NR | NR | 45.33 (31.43) | NR | NR | 25.58 (5.74) | NR | 1.44 (0.24) | NR | NR | 1.31 (0.06) | NR | NR |
| Yao et al. 2024 | 3 | 32 | Single-shell | 1000 | 32 | Semi-automated preprocessing (FSL) + manual ROI marking (rectangular, bilateral, projection & association fibers) | Single-center (Nanjing First Hospital, China) | 30 (14) | 60.83 (3.90) | PD-Early | 25 (13) | 63.04 (5.22) | NR | NR | 26.88 (8.91) | NR | NR | NR | 1.28 (0.46) | 26.52 (0.87) | NR | 1.45 (0.27) | 1.50 (0.11) | NR | NR | 1.61 (0.15) | NR | NR |
| Bae et al. 2023 (b) | 3 | 32 | Single-shell | 1000 | 32 | Semi-automated preprocessing (MATLAB analyzer) + manual ROI marking (bilateral, periventricular, SCR, SLF, SUF) | Single-center (Seoul National University Bundang Hospital, Korea) | 54 (23) | 69.0 (10.5) | PD | 54 (23) | 68.9 (9.4) | NR | NR | 16.6 (7.0) | NR | NR | NR | NR | 23.6 (4.7) | 26.5 (3.5) | NR | 1.51 (0.22) | NR | NR | 1.66 (0.20) | NR | NR |
| Pang et al. 2024 | 3 | 32 | Single-shell | 1000 | 64 | Semi-automated preprocessing (FSL) + manual ROI marking (5 mm spheres, bilateral, projection & association fibers) | Single-center (First Affiliated Hospital of China Medical University, China) | 35 (16) | 62.94 (5.22) | PD-MCI | 35 (18) | 65.77 (3.86) | NR | NR | 26.03 (7.61) | NR | NR | NR | 2.34 (1.00) | 21.20 (1.69) | 22.51 (1.62) | 4.11 (1.60) | 1.45 (0.26) | 1.53 (0.19) | 1.36 (0.32) | 1.50 (0.33) | 1.61 (0.18) | 1.38 (0.43) |
| Pang et al. 2024 | 3 | 32 | Single-shell | 1000 | 64 | Semi-automated preprocessing (FSL) + manual ROI marking (5 mm spheres, bilateral, projection & association fibers) | Single-center (First Affiliated Hospital of China Medical University, China) | 35 (16) | 62.94 (5.22) | PD-Dementia | 29 (16) | 63.87 (5.32) | NR | NR | 31.55 (8.96) | NR | NR | NR | 2.48 (1.02) | 11.03 (1.97) | 12.00 (3.15) | 4.83 (1.71) | 1.27 (0.21) | 1.16 (0.09) | 1.37 (0.29) | 1.50 (0.33) | 1.61 (0.18) | 1.38 (0.43) |
| Saito et al. 2023 | 3 | 12 | Single-shell | 1000 | 41 | Fully automated preprocessing (FSL + MRtrix3) + atlas-based ROI extraction (5 mm spheres, bilateral, projection & association fibers) | Multicenter initiative (4RTNI + FTLDNI, UCSF). | 17 (13) | 65.4 (6.0) | CBS | 21 (14) | 67.5 (5.7) | NR | NR | 31.0 (11.3) | NR | NR | NR | NR | NR | 25.0 (4.4) | NR | 1.51 (0.14) | 1.54 (0.23) | 1.48 (0.14) | 1.76 (0.04) | 1.76 (0.19) | 1.72 (0.14) |
| Shi et al. 2024 | 3 | 32 | Multi-shell | 10 ranging from 0 to 4000 | 120 | Semi-automated preprocessing (FSL) + atlas-based ROI extraction (5 mm spheres, bilateral, projection & association fibers) | Single-center (Liaocheng People’s Hospital, China) | 30 (10) | 55.8 (4.1) | MSA-P | 11 (5) | 56.6 (6.1) | NR | NR | NR | NR | NR | NR | NR | NR | NR | 3.33 (4.67) | 1.40 (0.15) | 1.43 (0.15) | 1.37 (0.16) | 1.63 (0.12) | 1.64 (0.14) | 1.62 (0.16) |
| Wang et al. 2024 | 3 | 15 | Single-shell | 0, 800 | 48 | Semi-automated preprocessing (FSL + JHU-ICBM Atlas) + atlas-based ROI extraction (5 mm spheres, bilateral, projection & association fibers) | Single-center (Ruijin Hospital, China) | 26 (9) | 62.73 (8.56) | PD | 71 (40) | 61.48 (9.72) | NR | NR | 26.45 (10.81) | NR | NR | NR | 1.67 (0.76) | 22.59 (4.66) | 26.96 (2.60) | 3.67 (3.03) | 1.27 (0.14) | NR | NR | 1.33 (0.14) | NR | NR |
| Wang et al. 2024 | 3 | 15 | Single-shell | 0, 800 | 48 | Semi-automated preprocessing (FSL + JHU-ICBM Atlas) + atlas-based ROI extraction (5 mm spheres, bilateral, projection & association fibers) | Single-center (Ruijin Hospital, China) | 26 (9) | 62.73 (8.56) | PD-NC | 21 (15) | 58.38 (9.59) | NR | NR | 24.81 (10.70) | NR | NR | NR | 1.50 (0.80) | 27.48 (1.08) | 28.90 (0.94) | 3.33 (3.18) | 1.29 (0.11) | NR | NR | 1.33 (0.14) | NR | NR |
| Wang et al. 2024 | 3 | 15 | Single-shell | 0, 800 | 48 | Semi-automated preprocessing (FSL + JHU-ICBM Atlas) + atlas-based ROI extraction (5 mm spheres, bilateral, projection & association fibers) | Single-center (Ruijin Hospital, China) | 26 (9) | 62.73 (8.56) | PD-MCI | 29 (18) | 61.03 (9.88) | NR | NR | 24.17 (10.81) | NR | NR | NR | 1.50 (0.78) | 23.28 (1.60) | 27.90 (1.40) | 3.33 (2.34) | 1.29 (0.13) | NR | NR | 1.33 (0.14) | NR | NR |
| Wang et al. 2024 | 3 | 15 | Single-shell | 0, 800 | 48 | Semi-automated preprocessing (FSL + JHU-ICBM Atlas) + atlas-based ROI extraction (5 mm spheres, bilateral, projection & association fibers) | Single-center (Ruijin Hospital, China) | 26 (9) | 62.73 (8.56) | PD-Dementia | 21 (7) | 65.19 (8.77) | NR | NR | 31.24 (9.81) | NR | NR | NR | 1.83 (0.40) | 16.76 (3.16) | 23.71 (1.90) | 5 (3.98) | 1.21 (0.16) | NR | NR | 1.33 (0.14) | NR | NR |
| Si et al. 2022 | 3 | 8 | Single-shell | 1000 | 30 | Semi-automated preprocessing (FSL) + manual ROI marking (5 mm spheres, projection & association fibers, left hemisphere only) | Single-center (Zhejiang University, China) | 129 (59) | 61.96 (7.21) | PD | 168 (96) | 59.85 (9.88) | NR | NR | 21.64 (12.37) | NR | NR | NR | NR | 22.08 (5.59) | 26.80 (3.83) | NR | 1.20 (0.17) | NR | NR | 1.31 (0.17) | NR | NR |
| Qin et al. 2023 | 3 | 12 | Single-shell | 1000 | 64 | Semi-automated preprocessing (FSL) + manual ROI marking (5 mm spheres, bilateral, projection & association & subcortical fibers, 3D Slicer) | Multi-center (PPMI international cohort) | 67 (43) | 60.10 (10.56) | PD | 153 (99) | 60.97 (9.47) | NR | NR | 20.76 (9.03) | NR | NR | 31.51 (13.46) | 1.58 | 27.56 (2.10) | NR | 0.57 (0.58) | 1.46 (0.24) | NR | NR | 1.55 (0.24) | NR | NR |
| Gu et al. 2023 | 3 | 8 | Single-shell | NR | NR | Semi-automated preprocessing (FSL) + manual ROI marking (5 mm spheres, left hemisphere, projection & association fibers) | Single-center (Zhejiang University Hospital, China) | 106 (40) | 60.30 (7.00) | PD | 124 (44) | 60.70 (7.25) | NR | NR | 22.82 (12.30) | NR | NR | NR | 2.30 (0.65) | NR | NR | 4.59 (4.74) | 1.22 (0.18) | NR | NR | 1.34 (0.19) | NR | NR |
| Gui et al. 2024 | 3 | 20 | Multi-shell | 1000, 2000 | 30 | Semi-automated preprocessing (FSL) + manual ROI marking (4 mm spheres, bilateral, projection, association & subcortical fibers) | Single-center (Suzhou Hospital of Nanjing Medical University, China) | 38 (18) | 70.34 (7.57) | PD | 49 (23) | 71.71 (7.49) | NR | 14.00 (6.67) | 25.98 (10.15) | NR | NR | NR | 2.51 (1.13) | 25.02 (2.93) | NR | 4.80 (3.09) | 1.47 (0.16) | NR | NR | 1.54 (0.18) | NR | NR |
| Gui et al. 2024 | 3 | 20 | Multi-shell | 1000, 2000 | 30 | Semi-automated preprocessing (FSL) + manual ROI marking (4 mm spheres, bilateral, projection, association & subcortical fibers) | Single-center (Suzhou Hospital of Nanjing Medical University, China) | 38 (18) | 70.34 (7.57) | PD-with insomnia | 28 | NR | NR | NR | NR | NR | NR | NR | NR | NR | NR | NR | 1.53 (0.16) | NR | NR | 1.54 (0.18) | NR | NR |
| Gui et al. 2024 | 3 | 20 | Multi-shell | 1000, 2000 | 30 | Semi-automated preprocessing (FSL) + manual ROI marking (4 mm spheres, bilateral, projection, association & subcortical fibers) | Single-center (Suzhou Hospital of Nanjing Medical University, China) | 38 (18) | 70.34 (7.57) | PD-without insomnia | 21 | NR | NR | NR | NR | NR | NR | NR | NR | NR | NR | NR | 1.42 (0.09) | NR | NR | 1.55 (0.18) | NR | NR |
| Gui et al. 2024 | 3 | 20 | Multi-shell | 1000, 2000 | 30 | Semi-automated preprocessing (FSL) + manual ROI marking (4 mm spheres, bilateral, projection, association & subcortical fibers) | Single-center (Suzhou Hospital of Nanjing Medical University, China) | 38 (18) | 70.34 (7.57) | PD-MCI | 24 | NR | NR | NR | NR | NR | NR | NR | NR | NR | NR | NR | 1.45 (0.15) | NR | NR | 1.55 (0.18) | NR | NR |
| Gui et al. 2024 | 3 | 20 | Multi-shell | 1000, 2000 | 30 | Semi-automated preprocessing (FSL) + manual ROI marking (4 mm spheres, bilateral, projection, association & subcortical fibers) | Single-center (Suzhou Hospital of Nanjing Medical University, China) | 38 (18) | 70.34 (7.57) | PD-NC | 25 | NR | NR | NR | NR | NR | NR | NR | NR | NR | NR | NR | 1.58 (0.12) | NR | NR | 1.55 (0.18) | NR | NR |
| Li et al. 2024 | 3 | NR | Single-shell | 1000 | 64 | Semi-automated preprocessing (FSL + DTI-TK) + atlas-based ROI extraction (5 mm spheres, bilateral, projection & association fibers) | Multi-center (PPMI international cohort) | 54 (32) | 60.6 (10.5) | PD | 114 (72) | 61.0 (9.5) | 5.2 (3.7) | 5.4 (4.0) | 21.3 (9.2) | NR | NR | 31.8 (13.4) | 1.6 (0.5) | 27.4 (2.1) | NR | 0.55 (0.52) | 1.64 (0.20) | 1.57 (0.18) | 1.74 (0.26) | 1.71 (0.21) | 1.64 (0.20) | 1.79 (0.25) |
| Li et al. 2024 | 3 | NR | Single-shell | 1000 | 64 | Semi-automated preprocessing (FSL + DTI-TK) + atlas-based ROI extraction (5 mm spheres, bilateral, projection & association fibers) | Multi-center (PPMI international cohort) | 54 (32) | 60.6 (10.5) | PD-Sleep Disorder | 43 (32) | 62.7 (9.8) | 6.0 (4.4) | 7.0 (4.2) | 22.3 (10.0) | NR | NR | 35.3 (15.7) | 1.6 (0.5) | 27.3 (2.3) | NR | 0.59 (0.57) | 1.59 (0.19) | 1.52 (0.18) | 1.70 (0.26) | 1.71 (0.21) | 1.64 (0.20) | 1.79 (0.25) |
| Li et al. 2024 | 3 | NR | Single-shell | 1000 | 64 | Semi-automated preprocessing (FSL + DTI-TK) + atlas-based ROI extraction (5 mm spheres, bilateral, projection & association fibers) | Multi-center (PPMI international cohort) | 54 (32) | 60.6 (10.5) | PD-Normal Sleep | 71 (40) | 60.0 (9.3) | 4.6 (3.0) | 4.5 (3.4) | 20.6 (8.6) | NR | NR | 29.7 (11.4) | 1.6 (0.5) | 27.7 (2.0) | NR | 0.52 (0.47) | 1.66 (0.19) | 1.60 (0.17) | 1.76 (0.26) | 1.71 (0.21) | 1.64 (0.20) | 1.79 (0.25) |
| Zhao et al. 2025 | 3 | 32 | Single-shell | 1000 | 128 | Semi-automated preprocessing (FSL + DSI Studio) + manual ROI marking (bilateral, projection & association fibers) | Single-center (Shandong Provincial Hospital, China) | 30 (11) | 59.2 (5.90) | PD | 51 (28) | 61.65 (8.27) | NR | NR | 29.60 (11.59) | NR | NR | NR | 2.5 | NR | 23.9 (4.38) | 4.08 (3.28) | 1.45 (0.17) | 1.46 (0.19) | 1.44 (0.18) | 1.64 (0.17) | 1.62 (0.17) | 1.66 (0.20) |
| Jiao et al. 2025 | NR | NR | Single-shell | 1000 | 64 | Semi-automated preprocessing (FSL) + atlas-based ROI extraction (JHU atlas, bilateral, projection & association fibers) | Single-center (Huashan Hospital, China (HPPI Database)) | 18 (8) | 60.83 (9.26) | PSP | 38 (27) | 68.55 (6.92) | NR | NR | 39.39 (16.43) | NR | NR | NR | NR | NR | NR | NR | 0.98 (0.16) | 1.03 (0.19) | 0.94 (0.17) | 1.16 (0.18) | 1.18 (0.23) | 1.31 (0.17) |
| Ren et al. 2025 | 3 | NR | Single-shell | 1000 | 64 | Semi-automated preprocessing (FSL) + manual ROI marking (5 mm spheres, bilateral, projection & association fibers, ICC validated) | Multi-center (PPMI international cohort) | 62 (39) | 60.03 (10.77) | PD | 139 (93) | 61.40 (9.83) | 2.5 (2.37) | 5 (4.49) | 21 (11.24) | NR | NR | NR | NR | 27.67 (2.25) | NR | 7 (8.99) | 1.49 (0.25) | NR | NR | 1.75 (0.32) | NR | NR |
| Shen et al. 2022 | 7 | NR | Multi-shell | 0, 1000, 3000 | NR | Semi-automated preprocessing (FSL + tractography) + manual ROI marking (5 mm spheres, bilateral, projection, association & subcortical fibers) | Single-center (Zhejiang University School of Medicine, China) | 47 (19) | 52.22 (8.84) | PD | 40 (21) | 54.55 (8.34) | 1.05 (2.06) | 8.85 (4.72) | 19.05 (11.28) | 2.03 (2.01) | NR | 31.55 (16.40) | 1.55 (0.45) | NR | 26.23 (3.18) | 5.22 (4.45) | 1.41 (0.26) | 1.39 (0.28) | 1.42 (0.24) | 1.50 (0.22) | 1.52 (0.22) | 1.48 (0.22) |
| Lin et al. 2025 | 3 | NR | Multi-shell | 200-1500 | 41 | Semi-automated preprocessing (FSL + QSM/SPM12) + manual ROI marking (3 mm spheres, bilateral, projection & association fibers) | Single-center (Fujian Medical University Union Hospital, China) | 81 (40) | 61.87 (7.49) | PD | 134 (81) | 63.71 (8.60) | NR | NR | 45.74 (17.23) | NR | NR | NR | 2.67 (0.37) | NR | NR | 7.33 (3.74) | 1.36 (0.24) | 1.36 (0.27) | 1.36 (0.20) | 1.53 (0.21) | 1.57 (0.19) | 1.48 (0.23) |
| Mareček et al., 2025 | 3 | 32 | Single-shell | 1000 | 31 | Automated atlas-based ROI extraction (JHU-ICBM CST & SLF, Snakemake pipeline) | Multi-center (First Faculty of Medicine, Charles University & General University Hospital, Czech Republic) | 48 (34) | 61.5 (9.9) | PD | 79 (48) | 59.5 (12.0) | 5.8 (4.3) | 7.5 (4.9) | 30.3 (13.1) | NR | NR | NR |  | 24.7 (3.1) | NR | 2.0 (1.8) | 1.43 (0.22) | NR | NR | 1.52 (0.21) | NR | NR |
| Ma et al., 2025 | 3 |  | Single-shell | 1000 | 31 | Semi-automated preprocessing (DTI Studio) + manual ROI marking (5 mm spheres, projection & association fibers, left hemisphere) | Single-center (Chinese Academy of Medical Sciences, China) | 33 (17) | 62.6 (6.1) | PD | 91 (39) | 65.2 (8.0) | 3.04 (2.16) | 12.51 (5.49) | 30.77 (11.93) | 2.36 (2.38) | NR | 49.52 (17.73) | 2.50 (0.75) | NR | 27.98 (2.13) | 7.65 (4.19) | 1.21 (0.18) | 1.22 (0.19) | 1.20 (0.18) | 1.28 (0.11) | 1.29 (0.11) | 1.27 (0.13) |

| **Supplementary Table 3.** Methodological Characteristics of Included Studies | | | | | | |
| --- | --- | --- | --- | --- | --- | --- |
| **Study** | **Diffusion acquisition** | **Preprocessing software** | **ROI Method** | **Type of ROI** | **Size of ROI (mm)** | **Center** |
| Ma et al. 2021 | single-shell | DTI Studio | manual | spheres/circular | 5.00 | single-center |
| Ruan et al. 2022 | multi-shell | DTI Studio | manual | spheres/circular | 4.00 | single-center |
| Si et al. 2022 | single-shell | FSL | manual | spheres/circular | 5.00 | single-center |
| Shen et al. 2022 | multi-shell | FSL | manual | spheres/circular | 5.00 | single-center |
| Bae et al. 2023 (a) | single-shell | DTI-ALPS analyzer | manual | spheres/circular | 5.00 | single-center |
| Bae et al. 2023 (b) | single-shell | MATLAB analyzer | manual | spheres/circular | Not reported | single-center |
| Cai et al. 2023 | single-shell | DTI Studio | manual | spheres/circular | 5.00 | single-center |
| Gu et al. 2023 | single-shell | FSL | manual | spheres/circular | 5.00 | single-center |
| Qin et al. 2023 | single-shell | FSL | manual | spheres/circular | 5.00 | multi-center |
| Georgiopoulos et al. 2024 | single-shell | MATLAB pipeline | manual | cube/rectangular | 2.00 | single-center |
| Gui et al. 2024 | multi-shell | FSL | manual | spheres/circular | 4.00 | single-center |
| Li et al. 2024 | single-shell | FSL / DTI-TK | atlas-based | spheres/circular | 5.00 | multi-center |
| Meng et al. 2024 | multi-shell | FSL | manual | spheres/circular | 4.00 | single-center |
| Wang et al. 2024 | single-shell | FSL | atlas-based | spheres/circular | 5.00 | single-center |
| Yao et al. 2024 | single-shell | FSL | manual | cube/rectangular | Not reported | single-center |
| Ni et al. 2025 | multi-shell | FSL/SPM | manual | spheres/circular | Not reported | single-center |
| Ren et al. 2025 | single-shell | FSL | manual | spheres/circular | 5.00 | multi-center |
| Zhao et al. 2025 | single-shell | FSL/DTI Studio | manual | Not reported | Not reported | single-center |
| Lin et al. 2025 | multi-shell | FSL/SPM | manual | spheres/circular | 3.00 | single-center |
| Mareček et al. 2025 | single-shell | FSL/MRtrix3 | atlas-based | Not reported | Not reported | multi-center |
| Ma et al. 2025 | single-shell | DTI Studio | manual | spheres/circular | 5.00 | single-center |

ff

| **Supplementary Table 4.** Quality assessment of included studies using the Newcastle–Ottawa Scale. This table summarizes the methodological quality of the included observational studies, assessed with the Newcastle–Ottawa Scale (NOS). The NOS evaluates three domains: Selection (maximum 4 points), Comparability (maximum 2 points), and Outcome (maximum 3 points). A star (*) represents one point, and a double star (★★) represents two points. The “Total score” column indicates the sum of points for each study (maximum score: 9). | | | | | | | | | |
| --- | --- | --- | --- | --- | --- | --- | --- | --- | --- |
| **Author/year** | **Selection** | **Selection** | | | | **Comparability** | **Outcome** | |  |
|  | **Representativeness of the sample** | **Representativeness of the sample** | **Sample size** | **Non-respondents** | **Ascertainment of the exposure (risk factor)** | **Control for important or additional factors** | **Assessment of the outcome** | **Statistical test** | **Total score** |
| Bae et al. 2023 (a) | ★ | ★ | - | - | ★★ | ★★ | ★★ | ★ | **8** |
| Bae et al. 2023 (b) | ★ | ★ | - | ★ | ★★ | ★★ | ★★ | ★ | **9** |
| Cai et al. 2023 | ★ | ★ | - | ★ | ★★ | ★★ | ★★ | ★ | **9** |
| Chen et al. 2021 | ★ | ★ | - | ★ | ★★ | ★★ | ★★ | ★ | **9** |
| Georgiopoulos et al. 2024 | ★ | ★ | - | ★ | ★★ | ★★ | ★★ | ★ | **9** |
| Gu et al. 2023 | ★ | ★ | - | ★ | ★★ | ★★ | ★★ | ★ | **9** |
| Gui et al. 2024 | ★ | ★ | - | ★ | ★★ | ★★ | ★★ | ★ | **9** |
| Hsu et al. 2024 | ★ | ★ | - | ★ | ★★ | ★★ | ★★ | ★ | **9** |
| Jiao et al. 2025 | ★ | ★ | - | ★ | ★★ | ★★ | ★★ | ★ | **9** |
| Li et al. 2024 | ★ | ★ | - | ★ | ★★ | ★★ | ★★ | ★ | **9** |
| Lin et al. 2025 | ★ | ★ | - | ★ | ★★ | ★★ | ★★ | ★ | **9** |
| Ma et al. 2021 | ★ | ★ | - | ★ | ★★ | ★★ | ★★ | ★ | **9** |
| Ma et al., 2025 | ★ | ★ | - | ★ | ★★ | ★★ | ★★ | ★ | **9** |
| Mareček et al., 2025 | ★ | ★ | - | ★ | ★★ | ★★ | ★★ | ★ | **9** |
| Meng et al. 2024 | ★ | ★ | - | ★ | ★★ | ★★ | ★★ | ★ | **9** |
| Ni et al. 2025 | ★ | ★ | - | ★ | ★★ | ★★ | ★★ | ★ | **9** |
| Ota et al. 2023 | ★ | ★ | - | ★ | ★ | ★★ | ★★ | ★ | **8** |
| Pang et al. 2024 | ★ | ★ | - | ★ | ★★ | ★★ | ★★ | ★ | **9** |
| Qin et al. 2023 | ★ | ★ | - | ★ | ★★ | ★★ | ★★ | ★ | **9** |
| Ren et al. 2025 | ★ | ★ | - | ★ | ★★ | ★★ | ★★ | ★ | **9** |
| Ruan et al. 2022 | ★ | ★ | - | ★ | ★★ | ★★ | ★★ | ★ | **9** |
| Saito et al. 2023 | ★ | ★ | - | ★ | ★★ | ★★ | ★★ | ★ | **9** |
| Shen et al. 2022 | ★ | ★ | - | ★ | ★★ | ★★ | ★★ | ★ | **9** |
| Shi et al. 2024 | ★ | ★ | - | ★ | ★★ | ★★ | ★★ | ★ | **9** |
| Si et al. 2022 | ★ | ★ | - | ★ | ★★ | ★★ | ★★ | ★ | **9** |
| Wang et al. 2024 | ★ | ★ | - | ★ | ★★ | ★★ | ★★ | ★ | **9** |
| Yao et al. 2024 | ★ | ★ | - | ★ | ★★ | ★★ | ★★ | ★ | **9** |
| Zhao et al. 2025 | ★ | ★ | - | ★ | ★★ | ★★ | ★★ | ★ | **9** |

| **Section and Topic** | **Item #** | **Checklist item** | **Location where item is reported** |
| --- | --- | --- | --- |
| **TITLE** | | |  |
| Title | 1 | Identify the report as a systematic review. | P1 |
| **ABSTRACT** | | |  |
| Abstract | 2 | See the PRISMA 2020 for Abstracts checklist. | P2 |
| **INTRODUCTION** | | |  |
| Rationale | 3 | Describe the rationale for the review in the context of existing knowledge. | P3 |
| Objectives | 4 | Provide an explicit statement of the objective(s) or question(s) the review addresses. | P3 |
| **METHODS** | | |  |
| Eligibility criteria | 5 | Specify the inclusion and exclusion criteria for the review and how studies were grouped for the syntheses. | P7-8 |
| Information sources | 6 | Specify all databases, registers, websites, organisations, reference lists and other sources searched or consulted to identify studies. Specify the date when each source was last searched or consulted. | P7-8 |
| Search strategy | 7 | Present the full search strategies for all databases, registers and websites, including any filters and limits used. | P7-8 |
| Selection process | 8 | Specify the methods used to decide whether a study met the inclusion criteria of the review, including how many reviewers screened each record and each report retrieved, whether they worked independently, and if applicable, details of automation tools used in the process. | P7-8 |
| Data collection process | 9 | Specify the methods used to collect data from reports, including how many reviewers collected data from each report, whether they worked independently, any processes for obtaining or confirming data from study investigators, and if applicable, details of automation tools used in the process. | P7-8 |
| Data items | 10a | List and define all outcomes for which data were sought. Specify whether all results that were compatible with each outcome domain in each study were sought (e.g. for all measures, time points, analyses), and if not, the methods used to decide which results to collect. | P7-8 |
|  | 10b | List and define all other variables for which data were sought (e.g. participant and intervention characteristics, funding sources). Describe any assumptions made about any missing or unclear information. | P7-8 |
| Study risk of bias assessment | 11 | Specify the methods used to assess risk of bias in the included studies, including details of the tool(s) used, how many reviewers assessed each study and whether they worked independently, and if applicable, details of automation tools used in the process. | P7-8 |
| Effect measures | 12 | Specify for each outcome the effect measure(s) (e.g. risk ratio, mean difference) used in the synthesis or presentation of results. | P7-9 |
| Synthesis methods | 13a | Describe the processes used to decide which studies were eligible for each synthesis (e.g. tabulating the study intervention characteristics and comparing against the planned groups for each synthesis (item #5)). | P7-8 |
|  | 13b | Describe any methods required to prepare the data for presentation or synthesis, such as handling of missing summary statistics, or data conversions. | P7-8 |
|  | 13c | Describe any methods used to tabulate or visually display results of individual studies and syntheses. | P7-8 |
|  | 13d | Describe any methods used to synthesize results and provide a rationale for the choice(s). If meta-analysis was performed, describe the model(s), method(s) to identify the presence and extent of statistical heterogeneity, and software package(s) used. | P7-8 |
|  | 13e | Describe any methods used to explore possible causes of heterogeneity among study results (e.g. subgroup analysis, meta-regression). | P7-8 |
|  | 13f | Describe any sensitivity analyses conducted to assess robustness of the synthesized results. | P7-8 |
| Reporting bias assessment | 14 | Describe any methods used to assess risk of bias due to missing results in a synthesis (arising from reporting biases). | P7-8 |
| Certainty assessment | 15 | Describe any methods used to assess certainty (or confidence) in the body of evidence for an outcome. | P7-8 |
| **RESULTS** | | |  |
| Study selection | 16a | Describe the results of the search and selection process, from the number of records identified in the search to the number of studies included in the review, ideally using a flow diagram. | P3-5 |
|  | 16b | Cite studies that might appear to meet the inclusion criteria, but which were excluded, and explain why they were excluded. | P3 |
| Study characteristics | 17 | Cite each included study and present its characteristics. | P3-5 |
| Risk of bias in studies | 18 | Present assessments of risk of bias for each included study. | P3-5 |
| Results of individual studies | 19 | For all outcomes, present, for each study: (a) summary statistics for each group (where appropriate) and (b) an effect estimate and its precision (e.g. confidence/credible interval), ideally using structured tables or plots. | P3-5 |
| Results of syntheses | 20a | For each synthesis, briefly summarise the characteristics and risk of bias among contributing studies. | P3-5 |
|  | 20b | Present results of all statistical syntheses conducted. If meta-analysis was done, present for each the summary estimate and its precision (e.g. confidence/credible interval) and measures of statistical heterogeneity. If comparing groups, describe the direction of the effect. | P3-5 |
|  | 20c | Present results of all investigations of possible causes of heterogeneity among study results. | P3-5 |
|  | 20d | Present results of all sensitivity analyses conducted to assess the robustness of the synthesized results. | P3-5 |
| Reporting biases | 21 | Present assessments of risk of bias due to missing results (arising from reporting biases) for each synthesis assessed. | P3-5 |
| Certainty of evidence | 22 | Present assessments of certainty (or confidence) in the body of evidence for each outcome assessed. | P3-5 |
| **DISCUSSION** | | |  |
| Discussion | 23a | Provide a general interpretation of the results in the context of other evidence. | P6-7 |
|  | 23b | Discuss any limitations of the evidence included in the review. |  |
|  | 23c | Discuss any limitations of the review processes used. |  |
|  | 23d | Discuss implications of the results for practice, policy, and future research. |  |
| **OTHER INFORMATION** | | |  |
| Registration and protocol | 24a | Provide registration information for the review, including register name and registration number, or state that the review was not registered. | Not applicable. |
|  | 24b | Indicate where the review protocol can be accessed, or state that a protocol was not prepared. | Not prepared. |
|  | 24c | Describe and explain any amendments to information provided at registration or in the protocol. | Not prepared. |
| Support | 25 | Describe sources of financial or non-financial support for the review, and the role of the funders or sponsors in the review. | Title page. |
| Competing interests | 26 | Declare any competing interests of review authors. | Title page. |
| Availability of data, code and other materials | 27 | Report which of the following are publicly available and where they can be found: template data collection forms; data extracted from included studies; data used for all analyses; analytic code; any other materials used in the review. | Title page. |

*From:*  Page MJ, McKenzie JE, Bossuyt PM, Boutron I, Hoffmann TC, Mulrow CD, et al. The PRISMA 2020 statement: an updated guideline for reporting systematic reviews. BMJ 2021;372:n71. doi: 10.1136/bmj.n71

For more information, visit: <http://www.prisma-statement.org/>
